# Supplementary material for: How do care home staff understand, manage and respond to agitation in people with dementia? A qualitative study
Source: BMJ Open. 2018 Jun 30;8(6):e022260. doi: 10.1136/bmjopen-2018-022260 (PMC6042579; doi:10.1136/bmjopen-2018-022260)
Supplement: Supplementary file 1 [file bmjopen-2018-022260supp001.pdf]

UCL DIVISION OF PSYCHIATRY
FACULTY OF BRAIN SCIENCES
BLOOMSBURY CAMPUS

Study to improve agitation and quality of life for people with dementia in care homes: Care worker semi-structured interview schedule

Introductions: As you know *I am a researcher from University College London and I will be conducting and recording this interview. Everything you say is confidential but I would like you to introduce yourselves for the recording so that the typist can identify you. My name is….*

Description of the research: *Thank you for agreeing to take part in this interview, I am interested in how people working in care homes think about and cope with residents with agitation; when talking about agitation we are referring to behaviours such as restlessness, pacing, shouting and verbal or physical aggression. We are asking because we know that this is something you and your colleagues manage a lot and we want to make use of your expertise. I will be asking about your experiences of working with people with agitation; about what works and what does not and what helps or hinders.*

We will use the information to help us to develop an intervention to reduce agitation in people with dementia living in care homes and we are interested in how to make the intervention something which is practical and fits with day to day work in busy care homes.

Confidentiality: *This interview is going to be audio recorded; whatever you tell me will be anonymised for the purposes of the study so you or any other individuals you mention will not be identifiable. However if you do disclose any information that you or someone else is being harmed I will ask your permission to disclose the information to my supervisor. We respect confidentiality but cannot keep it a secret if anyone is being seriously harmed.*

If at any point during the interview you feel that you need to stop or leave the room please do tell me.

Experiences of agitation:

I want you to think of a resident you know well who has been agitated. You don't need to tell me their name.

Q. Can you tell me a bit about the person and describe their behaviour and what happened?

- *What do you think may have caused their agitation?*
- *What do you think it means when they behave in this way?*
- *What else have you noticed in other residents when they become agitated?*

(Prompt: If there is nothing much to say about this resident ask them to think of someone else)

Managing agitation:

Q. When you think about the person and situation you were just describing, how did you manage the situation?

- *What was tried?*
- *What worked well?*
- *What did not work well?*
- *What did you feel able to do at the time?*
- *What did you notice other people doing?*
- *Tell me about good ways of communication with people like this?*
- *How do decisions get made about what to do?*
- *What else have you seen work well in other situations?*

- *How do you think ________________ would make a difference (pain control, talking, sensory stimulation, music, activities, more communication)*
- *What would stop them working? What would make them work?*

Barriers and facilitators
Q. What makes it easier for you to manage agitation in residents?

- *What is it about you?*
- *What is it about your role?*
- *What is it about the residents / their families?*
- *What is it about your team?*
- *What is it about your managers?*
- *What is it about your organisation / employer?*
- *What is it about the media e.g. TV and newspapers?*

Q. What makes it harder for you to manage agitation in residents?

- *What is it about you?*
- *What is it about your role?*
- *What is it about the residents / their families?*
- *What is it about your team?*
- *What is it about your managers?*
- *What is it about your organisation / employer?*
- *What is it about the media e.g. TV and newspapers?*

Impact of agitation
Q. What impact does resident's agitated behaviour have upon staff?
- *What effect does it have on how you feel at work?*
- *What effect does it have upon what you feel able to do?*
- *How does it affect your team?*

Support
Q. When it is difficult, do staff get support?
- *Do you feel you can ask for help when you need it?*
- *What sort of support do you find helpful? Who from?*
- *How do you and your colleagues support each other?*
- *What gets in the way of asking for support?*

Training
Q. What training have you had to help you to manage when residents become agitated?

(If they can think of training):
- *What was most useful?*
- *What was least useful?*

If they cannot think of past training):

Q. What training do you think would help you to feel more able to manage agitation?
- *What do you think would help you to put what you learn into practice?*
- *What would make it harder to put what you learn into practice?*

(Prompt re self, role, team, managerial, organisational factors)

Other
Q. Before we finish, is there anything else you would like to mention that we have not already covered?

Would you be willing to give us feed back about any materials we may develop in future?

Thank you for taking part today.
