# Supplementary material for: How do care home staff understand, manage and respond to agitation in people with dementia? A qualitative study
Source: BMJ Open. 2018 Jun 30;8(6):e022260. doi: 10.1136/bmjopen-2018-022260 (PMC6042579; doi:10.1136/bmjopen-2018-022260)
Supplement: Supplementary file 2 [file bmjopen-2018-022260supp002.pdf]

What works in practice? How do care home staff understand and manage agitation in people with dementia

We are writing to you because you were interviewed by us some time ago about your experiences of caring for residents with dementia who experience agitation. We wanted to thank you again for taking part in our study and also to send you a summary of what we found out.

We interviewed 25 members of staff in six different care homes about what happens when residents become agitated, how you as staff manage and respond, and what makes it harder or easier to manage.

We have used what you and the other staff we interviewed told us about what works to develop a staff training intervention which we are currently testing in ten care homes, to see if it helps staff when residents become agitated. We have tried to address some of the things that staff told us makes it harder to manage agitation and have included some of the ideas about what can make it easier.

What agitated behaviours did staff experience?

Staff told us that they were coping with a range of agitated behaviours, the most common ones were verbal and physical aggression, like hitting or pinching (especially during personal care), screaming and getting upset, and repetitive behaviours like wandering up and down or repeating words.

Many staff members told us that these behaviours were often quite extreme, could get worse very quickly, were hard to predict and could last a long time. Staff also told us that agitation was different for different each person and 'no two residents are the same'.

How did agitation make staff feel?

Many staff said it could be difficult when residents become agitated, especially if it involves more than one resident. Staff sometimes felt frightened, especially when residents were aggressive. When nothing seemed to help, staff felt hopeless and like they did not have power to make things better. Some staff talked about how even though they tried to stay calm and not react badly, sometimes they felt angry or upset when residents were very agitated.

What did staff feel caused agitation?

Staff described how although it can sometimes feel like a 'guessing game' when trying to work out why someone is agitated, there were often a number of possible reasons for what may be triggering agitation. These included:

- Agitation as a sign of being upset
- Agitation as a sign of physical pain or discomfort
- Agitation as caused by how other people react
- Agitation as caused by the persons surroundings e.g. too hot, noisy, boring
- Agitation as caused by the dementia itself

What helps staff to prevent agitation from happening?

All the staff we spoke to said it was important to get to know the individuals in their care and build trust with the residents. There were three main things that staff said helped them to get to know residents and prevented the residents from becoming agitated. These were:

1. **Seeing the person and not the disease**

> *Well, I think they should be able to come in, yes, do the personal care, but while you're doing the personal care, look at the rest of the person, not only the bit you're washing and dressing, remember that they're a human being, remember that they've lived a life. (Unit manager)*

2. **Connecting with previously valued identities**

> *Well, I always like to know what did you used to do in your time. What work do you like doing, you know. All the different things, really, in life… (Activities coordinator)*

3. **Playing along rather than correcting**

> *While you standing arguing with someone saying, no, you're 90 and your kids are all grown up, to them they're not. They're still at school. They need help so we just say to them... so just go in their reality with them. If they're looking for their kids, the kids are at school. They're all right. (Deputy Manager)*

4. **Making people feel comfortable and at home**

> *It's a 24 hour process and this is their home, they can get up when they like, as long as they eat and they feel comfortable, that's the most important. (Senior carer)*

How do staff respond when residents are agitated?

Staff described the strategies they used when residents were agitated and how they tried to be flexible and use 'trial and error':

> *There's no hard and fast rules, it's just really sort of trying to read the situation really. (Care assistant)*

Popular strategies included:

- Distracting and reassuring residents
- Giving agitated residents space
- Walking away

When these approaches did not work, staff would ask for help from senior colleagues and would sometimes get help from specialists outside of the care home. Some staff felt medication could help but said that often it had side effects and should only be used as a last resort.

What makes it harder or easier for staff to manage when residents are agitated?

1. Having the right approach makes it easier:

Staff described that being motivated, compassionate and caring, as well as recognising that you can only do your best, made it easier to do the job. They often compared this to feeling that some staff are 'only in it for the money'.

And what else gives us hope through the day? …I love my job, very much, and there is no explanation for that. You either have that or not. I could have done retail… I go through the day because I feel sorry for these people, simple as that. I… and I want to instinctively make it better. (Care assistant)

2. Communication is key:

Staff saw clear communication between team members as important. To respond well to agitation, staff needed to feel that they were not on their own and that colleagues would help if needed. Staff also said that building a good relationship with the families of residents was very important.

You do sometimes … think to yourself, I don't know if I can cope with this today but then… you take five minutes, you have a chat with one of the other carers and then we sort it out. (Care assistant)

3. Task focused vs person centred care:

Staff talked about not having enough time to spend with residents because of the staffing levels or because of all of the different jobs and tasks that they were expected to do during each shift. Staff said that they liked having 'hands on' managers who were approachable and that when they did not feel supported by management they would tend to just try and sort out problems themselves. Staff found it helpful to have good quality training but also said that learning on the job and from each other was very important.

I have had training [but] I've gone to management and they've taught me a different way to try and cope with it, I feel being there, dealing with it, doing it, is the best training. (Care assistant)

4. The care home industry makes it harder:

Staff talked about how television and newspapers always seem to focus on negative aspects of care, especially abuse and neglect, ignoring the good work that they saw happening and the impact of aggression from residents towards staff. This meant that families were more worried about their relatives living in a care home and staff felt this made them more worried about making mistakes or getting into trouble at work.

Sometimes it would be so lovely to hear a nice story about dementia, and staff, and what people do, and ... you don't hear things about residents lashing out at carers. (Care assistant)

Staff in all types of home talked about some of the financial problems in care homes and how they felt that focusing on business and profit made it harder for them to meet the needs of individuals.

Sometimes it can be challenging because if the budget doesn't meet ... then the staff need to be reduced, and ... the needs of the residents take second place. (Deputy Manager)

We would like to thank you again for taking part. We are also in the process of writing an article based on these findings so that a wide range of health and social care professionals will be able to learn from it.

If you have any thoughts or comments on this summary we would really appreciate your feedback. We are interested in hearing if you feel there is anything missing from this summary and whether it fits well or does not fit with your own experiences. You can either email xxx or write using the enclosed stamped addressed envelope.
