# Supplementary material for: How do care home staff understand, manage and respond to agitation in people with dementia? A qualitative study
Source: BMJ Open. 2018 Jun 30;8(6):e022260. doi: 10.1136/bmjopen-2018-022260 (PMC6042579; doi:10.1136/bmjopen-2018-022260)
Supplement: Supplementary file 4 [file bmjopen-2018-022260supp004.pdf]

Appendix 4: Table of themes

Overarching themes	Sub themes
1. Behaviours expressing unmet need	1.1 Unmet physical need<br>1.2 Unmet emotional need<br>1.3 Unmet environmental need
2. Staff emotional responses to agitation	2.1 Feeling powerless and disheartened<br>2.2 Feeling frightened<br>2.3 Trying not to react
3. Understanding the individual helps	3.1 Seeing the person not the disease<br>3.2 Connecting with previously valued identities<br>3.3 Playing along with rather than correcting<br>3.4 Making people feel comfortable and at home
4. Constraints on staff responses	4.1 Procedural constraints<br>4.2 Structural constraints<br>4.3 Support and training<br>4.4 Culture of fear and scrutiny
