# Supplementary material for: How do care home staff understand, manage and respond to agitation in people with dementia? A qualitative study
Source: BMJ Open. 2018 Jun 30;8(6):e022260. doi: 10.1136/bmjopen-2018-022260 (PMC6042579; doi:10.1136/bmjopen-2018-022260)
Supplement: Author's manuscript [file bmjopen-2018-022260.draft_revisions.pdf]

BMJ Open is committed to open peer review. As part of this commitment we make the peer review history of every article we publish publicly available.

When an article is published we post the peer reviewers' comments and the authors' responses online. We also post the versions of the paper that were used during peer review. These are the versions that the peer review comments apply to.

The versions of the paper that follow are the versions that were submitted during the peer review process. They are not the versions of record or the final published versions. They should not be cited or distributed as the published version of this manuscript.

BMJ Open is an open access journal and the full, final, typeset and author-corrected version of record of the manuscript is available on our site with no access controls, subscription charges or pay-per-view fees (http://bmjopen.bmj.com).

If you have any questions on BMJ Open's open peer review process please email info.bmjopen@bmj.com

BMJ Open

How do care home staff understand, manage and respond to agitation in people with dementia? A qualitative study

Journal:	*BMJ Open*
Manuscript ID	bmjopen-2018-022260
Article Type:	Research
Date Submitted by the Author:	08-Feb-2018
Complete List of Authors:	Rapaport, Penny; University College London, Division of Psychiatry<br>Livingston, Gill; UCL, Mental Health Science<br>Hamilton, Olivia; University College London, Division of Psychiatry<br>Turner, Rebecca; University College London, Division of Psychiatry<br>Stringer, Aisling; University College London, Division of Psychiatry<br>Robertson, Sarah; University College London, Division of Psychiatry<br>Cooper, Claudia; University College London
Keywords:	Dementia < NEUROLOGY, QUALITATIVE RESEARCH, Old age psychiatry < PSYCHIATRY

SCHOLARONE™
Manuscripts

For peer review only

How do care home staff understand, manage and respond to agitation in people with dementia? A qualitative study

Corresponding author:

Dr Penny Rapaport

Clinical Psychologist*

Tel: 0207 679 9716

Email: P.Rapaport@ucl.ac.uk

Authors:

Penny Rapaport[1,2](Mphil DclinPsy), Gill Livingston[1](MD FRCPsych), Olivia Hamilton[1](Msc), Rebecca Turner[1](MSc), Aisling Stringer[1](MSc), Sarah Robertson[1,2](PhD) and Claudia Cooper[1](PhD MRCPsych)

[1] UCL Department of Old Age Psychiatry, Division of Psychiatry, Wing A, Floor 6 Maple House, 149 Tottenham Court Rd, London, W1T 7NF, UK.

[2] North Thames CLAHRC

Word count: 3982

Tables: 3

Supplementary materials:

Appendix 1: Semi structured interview schedule
Appendix 2: Summary of results for participants
Appendix 3: SRQR checklist

For peer review only

ABSTRACT

Objectives: Little is known about how care home staff understand and respond to this distress in residents living with dementia labelled as "agitation". The aim of this study was to investigate care home staff experiences of caring for residents with dementia whom they perceive to be agitated; exploring how staff make sense of this agitation, how they respond, and what they find works or obstructs good practice.

Design: We conducted a qualitative semi-structured interview study using inductive thematic analysis to elicit themes.

Setting: We recruited staff from six English care homes including residential and nursing homes of differing sizes run by both the private and charity sector and located in urban and rural areas.

Participants: We interviewed 25 care home staff using purposive sampling to include staff of either sex, differing age, ethnicity, nationality and with different roles and experience.

Results: We identified four overarching themes: behaviours expressing unmet need; staff emotional responses to agitation; understanding the individual helps, and constraints on staff responses. Staff struggled with the paradox of trying to connect with the personhood of residents whilst seeing the person as separate to and, therefore, not responsible for their distress behaviours. Staff often felt powerless, frightened and overwhelmed and their responses were constrained by care home structures, processes and a culture of fear and scrutiny.

Conclusions: Future trials of psychosocial interventions for people with dementia living in care homes should support staff to identify and respond to residents' unmet needs and include how staff can look after themselves. Interventions should build upon the approaches and strategies already being used by care home staff in routine care and address constraints.

Key words: Dementia, agitation, care homes, psychosocial interventions, care staff

ARTICLE SUMMARY

Strengths and limitations of this study

- This is the first study to qualitatively explore how staff in care homes understand and respond to behaviours labelled as agitation in people living with dementia.
- Understanding how staff understand and manage agitation and what makes it harder or easier has informed the development of a psychosocial intervention that reflects and addresses the complexity of delivering interventions in care home settings.
- We recruited staff based upon manager's recommendations or existing research team and staff relationships which may have resulted in recruitment bias.
- We conducted semi-structured interviews rather than directly observing care interactions therefore our analysis reflects our interpretation of staff perspectives.

For peer review only

For peer review only - http://bmjopen.bmj.com/site/about/guidelines.xhtml

INTRODUCTION

Over 70% of UK care home residents have dementia,[1] often with multiple, complex needs and high levels of neuropsychiatric symptoms, including agitation.[2] While agitation is variously defined, the term is often used to refer to a range of behaviours; including restlessness, pacing, repetitive vocalisations and verbally or physically aggressive behaviour.[2 3] Agitation in care homes is associated with lower quality of life and higher care costs[4-6] and is persistent and distressing.[6 7] Being labelled as "agitated" may increase the difficulties individuals with dementia face by impacting upon "personhood"; how they are perceived, understood and managed by others.[8 9] This may increase prescribing of psychotropic medications, counter to the drive towards reducing the prescribing of antipsychotic medication for people with dementia and increased use of non-pharmacological interventions as an alternative.[10]

The Needs-driven, Dementia-compromised Behaviour (NDB) theory[11] posits that a wide number of different behaviours in dementia often labelled as "disruptive" arise from unmet needs. Needs may be emotional (communication, comfort or physical contact), recreational (stimulation, including touch and music; enjoyable activities) and physical (pain relief, thirst, hunger or treatment of constipation or infection). Environmental limitations can prevent needs being met, when staff are unavailable, unaware or inadequately skilled in communicating and interacting with people with dementia. In care homes, supervised interventions promoting better communication, interaction and understanding between care staff and people with dementia, can reduce behaviours defined as agitation for up to six months after the intervention.[12 13] Staff value interventions that encourage them to get to know, understand and connect with residents with dementia.[14]

Care home staff often have little training and are low paid, with high staff turnover.[15 16] Communication between staff and residents, for example, during personal care, can be dominated by instructions.[17] UK public policy calls for an "informed and effective workforce" to support people with dementia,[18] however, little is known about how front-line staff understand and respond to residents experiencing agitation. Knowing more about this may facilitate development of more effective interventions. This is the first qualitative study of care home staff experiences of caring for residents with dementia whom they perceive to be agitated. We explore how staff make sense of this agitation, how they respond, and what they find works or obstructs good practice.

METHODS

Setting and participants

We purposively selected care homes participating in MARQUE (Managing Agitation and Raising QUality of LifE in Dementia), a study involving people with dementia living in English care homes,[6] interviewing staff from varied care home settings: residential and nursing; differing sizes; private and charity sector; and in urban and rural areas. After contacting care home managers to explain the study, researchers who knew the staff approached individual staff members to explain the purpose of the study and give information sheets. If they wished to participate interviews were arranged at a convenient time. We sampled purposively to ensure that we interviewed staff of either sex, differing age, ethnicity, nationality and with different roles and experience.

For peer review only - http://bmjopen.bmj.com/site/about/guidelines.xhtml

Procedures

London (Queen's Square) National Research Ethics Service (NRES) committee gave approval (reference: 14/LO/0697). PR conducted interviews in private rooms in the care homes with staff who gave written, informed consent. We used a semi-structured interview schedule (see supplementary appendix 1) based on the literature, consultation with dementia family carers and research team expert opinion. After completing each set of interviews (in one care home) PR listened to the recordings, reflected on initial themes and revised the interview schedule as needed to incorporate new ideas expressed by care staff. We ceased interviews at thematic saturation, the point at which the authors' iterative discussions and reflections on additional interviews resulted in no further emergent themes. Individual interviews were digitally recorded and transcribed verbatim. Identifying information was removed to preserve anonymity; on completion of the analysis all recordings were deleted.

Data analysis

We used NVivo 11 software for qualitative analysis, taking an inductive thematic analytic approach.[19] PR and one of four independent raters (OH, RT, AS, SR) systematically coded each transcript into meaningful fragments and labelled these initial codes. Discrepancies were discussed and resolved. PR, GL and CC then organised the data into preliminary themes. We discussed the coding frames, using the constant comparison method, to identify similarities and differences in the data. We sought respondent validation by sending participants summaries of the findings, allowing them to comment on the accuracy and credibility of interpretations (see supplementary appendix 2). A checklist of methods used using the Standards for Reporting Qualitative Research (SRQR)[20] is presented in supplementary appendix 3.

RESULTS

Study participants

PR interviewed staff in six care homes in London (n=4), Kent (n=1) and Cambridge (n=1) between July 2014 and January 2015. Five homes were private and one was run by a charity. Three were nursing homes, two residential homes and one provided residential and nursing care. Table 1 summarises socio-demographic and employment characteristics of staff interviewed.

For peer review only

For peer review only - http://bmjopen.bmj.com/site/about/guidelines.xhtml

Table 1: Care staff socio-demographic and employment characteristics

Socio-demographic	Category	Care staff n (%)
Gender	Female<br>Male	17 (68)<br>8 (32)
Ethnicity	Asian or Asian British<br>Black or Black British<br>White British<br>White other<br>Mixed other	6 (24)<br>6 (24)<br>6 (24)<br>5 (20)<br>2 (8)
English as first language	No<br>Yes<br>Not known	13 (52)<br>11 (44)<br>1 (4)
Staff role	Care assistant<br>Senior carer<br>Manager / deputy manager<br>Team leader/unit manager<br>Activities coordinator<br>Nurse	9 (36)<br>5 (20)<br>5 (20)<br>2 (8)<br>2 (8)<br>2 (8)
Shift pattern	Days<br>Days and nights	18 (72)<br>7 (28)
Length of service	Less than 1 year<br>1 to 5 years<br>6 to 10 years	4 (16)<br>13 (52)<br>8 (32)
Nursing qualification	Yes<br>No	10 (40)<br>15 (60)

For peer review only

Qualitative findings

We identified four overarching themes: *Behaviours expressing unmet need*; *Staff emotional responses to agitation*; *Understanding the individual helps* and *Constraints on staff responses.* These are discussed below and presented in Table 2.

Table 2: Summary of main themes

Overarching themes	Sub themes
1. Behaviours expressing unmet need	1.1 Unmet physical need<br>1.2 Unmet emotional need<br>1.3 Unmet environmental need
2. Staff emotional responses to agitation	2.1 Feeling powerless and disheartened<br>2.2 Feeling frightened<br>2.3 Trying not to react
3. Understanding the individual helps	3.1 Seeing the person not the disease<br>3.2 Connecting with previously valued identities<br>3.3 Playing along with rather than correcting<br>3.4 Making people feel comfortable and at home
4. Constraints on staff responses	4.1 Procedural constraints<br>4.2 Structural constraints<br>4.3 Support and training<br>4.4 Culture of fear and scrutiny

Behaviours expressing unmet need

We describe a range of behaviours staff identified as being expressions of unmet, emotional, physical or environmental needs in Table 3. Staff experienced these behaviours as persistent,

For peer review only - http://bmjopen.bmj.com/site/about/guidelines.xhtml

unpredictable and often inter-related. The behaviours most commonly described were verbal and physical aggression expressed when staff provided intimate care, such as washing, dressing or assisting a resident to the toilet:

> *Like when you do the personal care he just goes with you for a while and then suddenly he'll react, it's like if you are not taking care of yourself the carers can be hit or sometimes those kinds of things, he'll just smash you like that. (Team leader; CH1)*

Table 3: Staff perceptions of unmet needs

Unmet need	Staff description
**Unmet physical need**	When we notice that the resident is confused, more confused might be a reason for a urine infection and otherwise incontinent residents, they are wet, we can see that their agitation is worse. (Team leader; CH1)<br><br>Like maybe they have pain in their legs, but they can't explain themselves. That is why I say you need patience, because they're shouting, they don't know where the pain is. (Senior carer; CH4)<br><br>We find that getting her to have a drink and something to eat, because sometimes, we think she's hungry and thirsty, but in her mind she's agitated and distressed for whatever reason. (Nurse; CH2)
**Unmet emotional need**	**Loss of independence:**<br>That he can't get up and do anything he wants. He's tried a couple of times, but we have to stop him, because he thinks, he remembers walking, but he doesn't know that… he can't walk, so we have to, like, plug his chair back or keep checking on him. (Care assistant 2; CH3)<br><br>**Shame and embarrassment:**<br>He's in a horizontal position, someone is trying to change his pad and he doesn't like that, because he doesn't like to be touched behind, for example, because he feels embarrassed. He would never realise how to express the embarrassment. He would kick and spit, but in his mind he's telling you, every bloody time. (Care assistant; CH4)<br><br>**Missing and protecting others:**<br>When you go to give care to another resident, she comes in and interferes, because she thinks that we are doing something to her relative, and she'll come and hit us, leave my sister alone, or, you know, leave my mother. (Nurse; CH2)<br><br>**Feeling insecure or abandoned:**<br>He feels he's been... I think he feels he's not wanted, he feels maybe they left him. I understand how he feels, how come I'm here, where are they, you know, I do understand. (Senior carer; CH4)
**Unmet environmental need**	They just receive very basic stimuli from the outside world, and if they don't like these stimuli, such as a scream, or a changing of lights, or a changing of position, or anything, they just, kind of, create turbulence, you know, in their status. (Care assistant; CH4)<br><br>Yes, we know, because like this one, who doesn't like any noise, he will stand up straightaway, like when he is having lunch, he will bang the cutlery on the table and then look at the person who is screaming or shouting. (Care assistant 2; CH5)

For peer review only - http://bmjopen.bmj.com/site/about/guidelines.xhtml

Unmet physical need

Many staff described how residents were often unaware or unable to communicate that they were in pain or unwell. Identifying the cause of distress involved a process of elimination and consideration of various factors such as hunger, thirst or medication. Some senior staff commented that these causes may be overlooked by care staff:

> *But you'll find a lot of… Someone might be shouting out and instead of asking, are you in pain, or investigating why, you'll find a lot of, well, I'll do you a cup of tea and that should do you for a little bit. (Deputy manager; CH2)*

Unmet emotional need

Staff drew on their knowledge of residents' past and present to try to understand how behaviours might arise from emotional distress. Some staff felt that physical needs were often viewed as more valid, real or more deserving of staff responses than emotional needs, especially when staff were under pressure:

> *Unfortunately, what it comes to is you start to think to yourself, well, these people have real needs… because she wants the companionship, but, you know, we do have a hierarchy in terms of- is the person pain free, are they well hydrated, are they fed well, are they comfortable, and then maybe you can get to the social needs. (Deputy manager; CH2)*

Some staff perceived overt expressions of distress such as repeated screaming or calling out as attention seeking or intentionally demanding:

> *It's a bit of a game sometimes for him I think. There's a lot of play-acting getting involved. This guy probably has lot more capacity than he thinks. (Care assistant; CH3)*

Unmet environmental need

Staff described how residents may be distressed due to a lack of interaction and stimulation:

> *Just the whole idea of them sitting in a chair is no good. I don't like that. The brain must be stimulated, even if it is a small amount it must be stimulated. (Care assistant; CH3)*

Staff considered that care home environments contributed to unmet needs:

> *I mean, if you walk into the lounge in a care home it isn't like your home. There isn't a sofa, there's single chairs, and who has single chairs? And something that small can make a big part on someone, especially if someone is affectionate; they want to sit next to somebody. (Care assistant; CH2)*

A number of staff described residents feeling trapped, evoking images of imprisonment:

> *Well, there is one. Like xxx. He find that this home - can I use that word? Prison…He finds this home that it's a prison. (Activities coordinator; CH3)*
>
> *I can open the door. I can have a walk outside. It's not for them. They are always going, either in this left corridor, or to the far end of the right one, or in the lounge, or in the dining area. That's it. Finished. (Care assistant; CH4)*

For peer review only - http://bmjopen.bmj.com/site/about/guidelines.xhtml

Staff emotional responses to agitation

Staff sometimes struggled to respond to residents' behaviours, especially if the behaviour involved more than one resident. They reported being unable to give space to a resident or let them express discomfort, as they wanted to minimise the impact on others:

> *So it can be very difficult if he shouts all night. It's not fair on them because he's disrupting somebody and they don't sleep. Alright fair enough, he's got his got his own problems but what about the other residents. (Senior carer; CH4)*

Feeling powerless and disheartened

Staff frequently described feeling powerless, especially when attempts to alleviate a resident's distress were not working, or if they could not make sense of a resident's behaviour. This was particularly so when residents expressed repetitive behaviours that persisted over time.

> *But to have someone distressed in front of you, then… someone else is getting distressed… They are looking at you to try and do something, and you can't do anything. (Deputy manager; CH5)*

At these times staff judged themselves as not 'doing a good job' and feeling judged by others undermining their identity as caring professionals.

> *It can make you feel sometimes, when things aren't working, that you've failed… sometimes you do go home disarmed, because you feel that you haven't been able to do the best for that person. (Activities coordinator; CH2)*

Feeling frightened

Staff also spoke about feeling frightened of being harmed. This connected to feelings of powerlessness, especially when residents hit or shouted at staff. This anticipation of harm affected how staff approached and responded to residents:

> *They are scared. It doesn't mean they don't do it, but, you know what I mean? While you're doing things, you're not doing with all the openness and things; you do it with an 'oohf'. (Care assistant; CH4)*

Staff narrated these behaviours as 'part of the job', yet highlighted how difficult it was fearing for their own safety:

> *Sometimes it is quite traumatic to be slapped or to be kicked or to be scratched or... you know, it's not an easy thing to say, okay, I'll brush it off. (Deputy manager; CH5)*

Trying not to react

Although staff described resident behaviours as unintentional they sometimes reacted in ways they regretted. They described trying 'not to react' to aggression, the effort required to stay calm and how their immediate, instinctual reactions could at times, escalate behaviours.

> *It may make you react in a way that you don't want to, because you know these residents can't help their behaviours, but… you're stressed... and you may say something... you shouldn't say, or... raise your voice at a resident, which you... shouldn't do, but at that moment, you're thinking, oh, no, again. (Nurse; CH2)*

For peer review only - http://bmjopen.bmj.com/site/about/guidelines.xhtml

Understanding the individual helps

Staff found that having time to get to know and understand residents was critical to building trust and familiarity with residents. This helped them to understand and respond to residents' needs.

Seeing the person not the disease

Staff described what they termed a "person-centred approach" as getting to know the person with dementia informing their responses to distress behaviours. They talked about seeing residents as equals and imagining how they would feel in their situation. Staff drew upon notions of shared humanity to describe how they maintained empathy and compassion, connecting with the "personhood" of individuals. However, staff also described behaviours that may be construed as socially unacceptable, like swearing or displaying aggression, as part of the dementia and separate to the person, moving between these different, arguably contradictory positions. This tension was apparent when staff described attempts to talk directly to residents about behaviours staff considered unacceptable. Seeing people with dementia as like themselves led some to feel that they might be able to control their behaviour and were, therefore, to blame for it.

> *We said that you have to apologise to your wife because it was not nice ... swearing at her. So ... maybe he realise but he say, I don't want to, I'm not going to apologise... Maybe he just doesn't remember… when he was swearing. (Care assistant; CH5)*

Connecting with previously valued identities

Knowing about a person's past and using this during care was viewed as a respectful way to calm residents. Sometimes relatives facilitated this process by sharing information and explaining 'what works'. Staff often perceived behaviours as expressing distress at losing independence and spoke of supporting individuals to reconnect with what was important to them.

> *She likes to look nice... sometimes... [we] sit her down in her bedroom, and… encourage her to put makeup on… her doing it can… help settle her. (Unit manager; CH3)*

> *[It] is a way of showing your independence… So giving her the money that she can pay with, she feels that she's paid... and that... she's worthy to have that sort of thing. (Activities coordinator; CH2)*

Playing along rather than correcting

Staff struggled to know how to respond when residents were disoriented, especially when residents were unaware of the extent of their dependency. Staff at all levels talked about "playing along" or "entering their reality" as better than trying to orient people. This was not simple and did not always have a positive effect. Staff found it hard to decide when to stop "playing along". They felt uncomfortable 'lying' to residents as it could increase confusion.

> *While you standing arguing with someone saying, no, you're 90 and your kids are all grown up. To them they're … still at school...You wouldn't go as far as saying, oh, they've just gone to the shop. They'll be back in a minute because then that minute they could still be, well, where is she? (Deputy manager; CH6)*

Making people feel comfortable and at home

Making people feel at home involved creating a stimulating and comforting environment. Staff described trying to comfort residents, particularly more impaired residents, using music, touch and other sensory stimulation, in addition to 'activities' led by specialist staff. They talked about how 'touch' made a big difference to residents, otherwise only touched during personal care.

For peer review only - http://bmjopen.bmj.com/site/about/guidelines.xhtml

I'll say to him, do you want to dance? Because he liked to dance. He'll take me really close and we'll have a little bit of a dance. (Care assistant; CH2)

Constraints on staff responses

Procedural constraints

As noted above, getting to know the residents and delivering "person-centred" care and was often described as a preferred approach. Although a person-centred approach was valued, many acknowledged that care delivery was often divided into a series of tasks, with an inherent tension between task-focused and person-focused approaches.

Changing in that [person-centred] direction is very difficult, because people start thinking, oh, if I do that, I might get told off. If I do that, then I won't be able to fill in the dishwasher by quarter past 11, or if I do this instead of that, then they're going to tell me off because I didn't take the bin, so it's all this kind of balancing act. (Deputy manager; CH5)

Additionally, there seemed to be an implicit hierarchy of how staff should respond to resident's needs. This meant that they would prioritise 'basic needs' over a need for company or interaction. This hierarchy of how staff should be ordering their time was often discussed in relation to care staff not feeling that they have the time to engage residents in activities themselves, relying on activities coordinators for this:

And you might be doing an activity with someone, the guy in one of the rooms pressed the emergency, we have a guy who presses the…he is almost like needs one to one care so you might be rushed off to attend to him really. It really is, the activities really does demand an extra carer I think. (Care assistant; CH3)

Structural constraints

Staff in all homes commented on financial challenges facing the sector, describing a business culture incompatible with delivering personalised care, particularly when it reduced staffing levels and therefore time. Staff also spoke about how, in a home that was part of a larger company, they felt anonymous and disconnected from the wider organisation. One staff member recalled having her glasses broken by a resident and the company refusing to pay for repair. Other staff said that minimum wage pay, anti-social shift patterns and staffing levels make it harder to maintain compassion:

Sometimes it can be challenging because if the budget doesn't meet... then the staff need to be reduced, and... The needs of the residents take second place. (Deputy manager; CH5)

And, I think, with these big homes where there are 109, 110 beds, it's too much... It is just a conveyor belt; who's next, who's next, who's next? (Deputy manager; CH6)

Support and training

Staff described feeling devalued by managers and not heard or taken seriously when they raised concerns. Where they felt unappreciated by residents and relatives, appreciation by managers took on additional importance.

You don't always feel valued for the job that you're doing; it is a very difficult job. It does have an effect... on your working practise… caring for people all day and it doesn't always feel as though staff are really cared for. (Unit manager; CH3)

For peer review only - http://bmjopen.bmj.com/site/about/guidelines.xhtml

In most homes staff described how they would “keep it to ourselves”, seeking support from their immediate team, assuming that managers would be unhelpful. This response was perhaps heightened by feeling that managers cannot understand what they are experiencing as front-line carers and will not help find solutions:

> *Well, even if I told somebody, I don’t know what they could do. What could they do? (Care assistant 2; CH3)*

Although many care staff spoke of a lack of managerial support, they (and the managers) also highlighted examples of good practice. Being ‘hands on’ connected to staff feeling that managers could understand their difficulties, and had done or could do the jobs themselves. Staff also highlighted how learning from peers and senior staff through discussion, observation and joint working enabled them to find new ways to respond to resident distress.

> *I have had training [but] I’ve gone to management and they’ve taught me a different way to try and cope with it, I feel being there, dealing with it, doing it, is the best training. (Care assistant; CH2)*

Culture of fear and scrutiny

Staff felt that the media focussed on negative aspects of care, particularly abuse and neglect. They thought the media overlooked the good practice that they saw, as well as the impact, particularly of behaviours that in other contexts would be construed as abusive, from residents towards staff. This eroded staff morale and motivation.

> *Sometimes it would be so lovely to hear a nice story about dementia, and staff, and what people do, and... You don’t hear things about residents lashing out at carers. (Care assistant; CH2)*

In some cases, negative perceptions of care homes cultivated a culture of fear of making mistakes or getting into trouble. This stifled more creative and flexible approaches to meeting resident’s unmet needs. Staff felt that appearances were sometimes prioritised over minimising distress, for example, insisting a resident changed a dirty top or came out of their room even when this would increase distress.

> *There’s the cover your back kind of fear to people… I think that translates back into the negative thing where you don’t want to try a new thing in case it hurts someone or it puts them at risk (Deputy manager; CH2)*

> *And they’ll say, why is my mum being in bed? And, you know… obviously we tried our best and… it does annoy. We’re always writing it down and just inform the Nurse so we don’t get in trouble (Care assistant 2; CH4)*

DISCUSSION

Main findings

To our knowledge, this is the first qualitative study to explore how care home staff experience, understand and respond to behaviours labelled as agitation in people with dementia and what helps or hinders their responses. We identified four overarching themes: *Behaviours expressing unmet need*; *Staff emotional responses to agitation*; *understanding the individual helps* and *constraints on staff responses*. Staff described a range of behaviours as expressions of unmet needs and wanted to

For peer review only - http://bmjopen.bmj.com/site/about/guidelines.xhtml

deliver person-centred care, but struggled to do this when feeling overwhelmed, unsupported by management, and unsafe or fearful. They faced tensions in deciding how far to go along with a resident's disorientation or how to separate a person from their behaviour without undermining personhood.

Although they knew their response could alleviate distress they were not always able to do so. As a result they felt powerless and frightened, impacting upon their capacity to respond. These findings are consistent with an existing study that found behaviours perceived as aggressive, uncooperative and unpredictable were felt to be most difficult to manage.[21] When 'trying not to react' in unhelpful ways and to meet resident unmet needs, staff were balancing their own safety, the needs and safety of other residents and those of the resident.

The impact of structural and procedural factors upon staff wellbeing and care practices has previously been documented qualitatively[22-25] and quantitatively.[16 26] Consistent with this, staff here indicated that they internalised a culture of scrutiny and fear from within and outside of care homes. This sometimes prevented staff from trying new approaches and staff felt that the care home sector was increasingly incompatible with an individualised approach to meeting resident needs. This is concerning given that inappropriate treatment of people with dementia in residential and day care often occurs when staff feel unable to meet clients' needs.[27]

Clinical implications

We have built our findings into the MARQUE intervention, and are currently testing it in a randomised controlled trial. The intervention conceptualises behaviours as expressions of unmet needs in residents and introduces heuristics and practical activities, focused upon staff developing an in-depth understanding of the resident's distress. It makes concepts accessible by using real-life examples and suggestions and is easy to implement. Many strategies highlighted as useful in this study have been incorporated, such as: using tools to get to know residents better; finding ways to offer comfort during routine care; allowing time for pressured staff to communicate with one another and find ways to manage their own feelings. External barriers to putting potentially beneficial strategies into practice have been addressed by delivering the intervention to all staff, involving managers, developing action plans, encouraging staff to try out and reflect upon new approaches, offering ongoing supervision, and leaving materials for new staff.

Limitations

Although our sampling meant that we accessed a breadth of viewpoints, contributing to the richness of this account, we directly approached staff based upon manager's recommendations or existing research team and staff relationships. There, therefore, may be an inherent recruitment bias. In relying on interviews with care home staff, we present their narration of their experiences and perceptions of how they deliver care. We must be cautious not to overgeneralise our findings. Without observing care interactions directly we are unable to triangulate this with what happens in practice or with resident experiences.

Future research

This study highlights the complex interrelationship between notions of personhood and needs-driven behaviours labelled as agitation[8] building upon research highlighting high skill levels required by staff expected to deliver person centred care in care homes, and the complexity of achieving

For peer review only - http://bmjopen.bmj.com/site/about/guidelines.xhtml

this.[23] Future research should explore the long term impact of interventions on care home culture and if and how they become embedded in care home practices.

For peer review only

For peer review only - http://bmjopen.bmj.com/site/about/guidelines.xhtml

Author's contributions

All authors made a substantial contribution to this work. PR, CC and GL all contributed to the conception and design of the study and PR drafted the paper. All authors critically revised it and gave final approval for this version to be published. PR collected all the data and coded all the interview transcripts. OH, RT, AS, SR all coded some of the interview transcripts. PR, CC and GL then organised the data into preliminary themes.

Funding

This study was funded by a grant from the UK Economic and Social Research Council and the National Institute of Health Research Grant number NIHR/ESRC ES/L001780/1. Drs Cooper and Livingston are supported by the UCLH NIHR Biomedical Research Centre. This research was supported by the National Institute for Health Research (NIHR) Collaboration for Leadership in Applied Health Research and Care North Thames at Bart's Health NHS Trust (NIHR CLAHRC North Thames).

The views expressed are those of the author(s) and not necessarily those of the NHS, the NIHR or the Department of Health. The funders had no role in study design, data collection and analysis, decision to publish, or preparation of the manuscript.

Competing interests

The authors declare that they have no competing interests.

Data Sharing statement

No additional data are available.

Acknowledgments

We wish to thank all the care home staff who participated in this study and all the Alzheimer's Society research network volunteers for their contribution. Thank you to Joanna Murray for her contribution to the qualitative analysis.

For peer review only

For peer review only - http://bmjopen.bmj.com/site/about/guidelines.xhtml

REFERENCES

1. Prince M, Knapp M, Guerchet M, et al. Dementia UK: update. *Alzheimer's Society, London* 2014
2. Cohen-Mansfield J. Conceptualization of agitation: results based on the Cohen-Mansfield agitation inventory and the agitation behavior mapping instrument. *International Psychogeriatrics* 1997;8(S3):309-15.
3. Cummings J, Mintzer J, Brodaty H, et al. Agitation in cognitive disorders: International Psychogeriatric Association provisional consensus clinical and research definition. *International Psychogeriatrics* 2015;27(01):7-17.
4. Morris S, Patel N, Baio G, et al. Monetary costs of agitation in older adults with Alzheimer's disease in the UK: prospective cohort study. *BMJ open* 2015;5(3) doi: 10.1136/bmjopen-2014-007382
5. Okura T, Plassman BL, Steffens DC, et al. Prevalence of neuropsychiatric symptoms and their association with functional limitations in older adults in the United States: the aging, demographics, and memory study. *Journal of the American Geriatrics Society* 2010;58(2):330-37.
6. Livingston G, Barber J, Marston L, et al. Prevalence of and associations with agitation in residents with dementia living in care homes: MARQUE cross-sectional study. *British Journal of Psychiatry Open* 2017;3(4):171-78.
7. Savva GM, Zaccai J, Matthews FE, et al. Prevalence, correlates and course of behavioural and psychological symptoms of dementia in the population. *The British Journal of Psychiatry* 2009;194(3):212-19. doi: 10.1192/bjp.bp.108.049619
8. Higgs P, Gilleard C. Interrogating personhood and dementia. *Aging & Mental Health* 2015:1-8.
9. Kitwood T, Bredin K. Towards a theory of dementia care: personhood and well-being. *Ageing and society* 1992;12(03):269-87.
10. Banerjee S. The use of antipsychotic medication for people with dementia: Time for action. London: Institute of Psychiatry: King's college London, 2014.
11. Algase DL, Beck C, Kolanowski A, et al. Need-driven dementia-compromised behavior: An alternative view of disruptive behavior. *American Journal of Alzheimer's Disease and Other Dementias* 1996;11(6):10-19.
12. Livingston G, Kelly L, Lewis-Holmes E, et al. Non-pharmacological interventions for agitation in dementia: systematic review of randomised controlled trials. *The British Journal of Psychiatry* 2014;205(6):436-42.
13. Jutkowitz E, Brasure M, Fuchs E, et al. Care-Delivery Interventions to Manage Agitation and Aggression in Dementia Nursing Home and Assisted Living Residents: A Systematic Review and Meta-analysis. *J Am Geriatr Soc* 2016;64(3):477-88. doi: 10.1111/jgs.13936
14. Rapaport P, Livingston G, Murray J, et al. Systematic review of the effective components of psychosocial interventions delivered by care home staff to people with dementia. *BMJ open* 2017;7(2):e014177.
15. Franklin B. The future care workforce: London: ILC-UK, 2014.
16. Testad I, Mikkelsen A, Ballard C, et al. Health and well-being in care staff and their relations to organizational and psychosocial factors, care staff and resident factors in nursing homes. *International journal of geriatric psychiatry* 2010;25(8):789-97.
17. Cohen-Mansfield J, Creedon MA, Malone T, et al. Dressing of cognitively impaired nursing home residents: Description and analysis. *The Gerontologist* 2006;46(1):89-96.
18. Department of Health. Living well with Dementia: A National Dementia Strategy., 2009.
19. Braun V, Clarke V. Using thematic analysis in psychology. *Qualitative Research in Psychology* 2006;3(2):77-101.
20. O'Brien BC, Harris IB, Beckman TJ, et al. Standards for reporting qualitative research: a synthesis of recommendations. *Academic Medicine* 2014;89(9):1245-51.
21. Brodaty H, Draper B, Low LF. Nursing home staff attitudes towards residents with dementia: strain and satisfaction with work. *Journal of advanced nursing* 2003;44(6):583-90.

For peer review only

For peer review only - http://bmjopen.bmj.com/site/about/guidelines.xhtml

22. Killett A, Hyde P, Burns D, et al. How organizational factors interact to influence the quality of care of older people in the care home sector. *Journal of Health Services Research & Policy* 2013;18(1_suppl):14-22.
23. Stanyon MR, Griffiths A, Thomas SA, et al. The facilitators of communication with people with dementia in a care setting: an interview study with healthcare workers. *Age and Ageing* 2016;45(1):164-70. doi: 10.1093/ageing/afv161
24. Lawrence V, Fossey J, Ballard C, et al. Helping staff to implement psychosocial interventions in care homes: Augmenting existing practices and meeting needs for support. *International Journal of Geriatric Psychiatry* 2016;31(3):284-93. doi: http://dx.doi.org/10.1002/gps.4322
25. Backhouse T, Killett A, Penhale B, et al. The use of non-pharmacological interventions for dementia behaviours in care homes: findings from four in-depth, ethnographic case studies. *Age and Ageing* 2016;45(6):856-63. doi: 10.1093/ageing/afw136
26. Vernooij-Dasssen MJ, Faber MJ, Olde Rikkert MG, et al. Dementia care and labour market: the role of job satisfaction. *Aging and Mental Health* 2009;13(3):383-90.
27. Sormunen S, Topo P, Eloniemi-Sulkava U, et al. Inappropriate treatment of people with dementia in residential and day care. *Aging and mental health* 2007;11(3):246-55.

For peer review only

For peer review only - http://bmjopen.bmj.com/site/about/guidelines.xhtml

UCL DIVISION OF PSYCHIATRY
FACULTY OF BRAIN SCIENCES
BLOOMSBURY CAMPUS

Study to improve agitation and quality of life for people with dementia in care homes: Care worker semi-structured interview schedule

Introductions: As you know *I am a researcher from University College London and I will be conducting and recording this interview. Everything you say is confidential but I would like you to introduce yourselves for the recording so that the typist can identify you. My name is….*

Description of the research: *Thank you for agreeing to take part in this interview, I am interested in how people working in care homes think about and cope with residents with agitation; when talking about agitation we are referring to behaviours such as restlessness, pacing, shouting and verbal or physical aggression. We are asking because we know that this is something you and your colleagues manage a lot and we want to make use of your expertise. I will be asking about your experiences of working with people with agitation; about what works and what does not and what helps or hinders.*

We will use the information to help us to develop an intervention to reduce agitation in people with dementia living in care homes and we are interested in how to make the intervention something which is practical and fits with day to day work in busy care homes.

Confidentiality: *This interview is going to be audio recorded; whatever you tell me will be anonymised for the purposes of the study so you or any other individuals you mention will not be identifiable. However if you do disclose any information that you or someone else is being harmed I will ask your permission to disclose the information to my supervisor. We respect confidentiality but cannot keep it a secret if anyone is being seriously harmed.*

If at any point during the interview you feel that you need to stop or leave the room please do tell me.

Experiences of agitation:
I want you to think of a resident you know well who has been agitated. You don't need to tell me their name.

Q. Can you tell me a bit about the person and describe their behaviour and what happened?
- *What do you think may have caused their agitation?*
- *What do you think it means when they behave in this way?*
- *What else have you noticed in other residents when they become agitated?*

(Prompt: If there is nothing much to say about this resident ask them to think of someone else)

Managing agitation:
Q. When you think about the person and situation you were just describing, how did you manage the situation?
- *What was tried?*
- *What worked well?*
- *What did not work well?*
- *What did you feel able to do at the time?*
- *What did you notice other people doing?*
- *Tell me about good ways of communication with people like this?*
- *How do decisions get made about what to do?*
- *What else have you seen work well in other situations?*

For peer review only - http://bmjopen.bmj.com/site/about/guidelines.xhtml

- *How do you think _________________ would make a difference (pain control, talking, sensory stimulation, music, activities, more communication)*
- *What would stop them working? What would make them work?*

Barriers and facilitators

Q. What makes it easier for you to manage agitation in residents?

- *What is it about you?*
- *What is it about your role?*
- *What is it about the residents / their families?*
- *What is it about your team?*
- *What is it about your managers?*
- *What is it about your organisation / employer?*
- *What is it about the media e.g. TV and newspapers?*

Q. What makes it harder for you to manage agitation in residents?

- *What is it about you?*
- *What is it about your role?*
- *What is it about the residents / their families?*
- *What is it about your team?*
- *What is it about your managers?*
- *What is it about your organisation / employer?*
- *What is it about the media e.g. TV and newspapers?*

Impact of agitation

Q. What impact does resident's agitated behaviour have upon staff?

- *What effect does it have on how you feel at work?*
- *What effect does it have upon what you feel able to do?*
- *How does it affect your team?*

Support

Q. When it is difficult, do staff get support?

- *Do you feel you can ask for help when you need it?*
- *What sort of support do you find helpful? Who from?*
- *How do you and your colleagues support each other?*
- *What gets in the way of asking for support?*

Training

Q. What training have you had to help you to manage when residents become agitated?

(If they can think of training):

- *What was most useful?*
- *What was least useful?*

If they cannot think of past training):

Q. What training do you think would help you to feel more able to manage agitation?

- *What do you think would help you to put what you learn into practice?*
- *What would make it harder to put what you learn into practice?*

(Prompt re self, role, team, managerial, organisational factors)

Other

Q. Before we finish, is there anything else you would like to mention that we have not already covered?

Would you be willing to give us feed back about any materials we may develop in future?

Thank you for taking part today.

For peer review only - http://bmjopen.bmj.com/site/about/guidelines.xhtml

What works in practice? How do care home staff understand and manage agitation in people with dementia

We are writing to you because you were interviewed by us some time ago about your experiences of caring for residents with dementia who experience agitation. We wanted to thank you again for taking part in our study and also to send you a summary of what we found out.

We interviewed 25 members of staff in six different care homes about what happens when residents become agitated, how you as staff manage and respond, and what makes it harder or easier to manage.

We have used what you and the other staff we interviewed told us about what works to develop a staff training intervention which we are currently testing in ten care homes, to see if it helps staff when residents become agitated. We have tried to address some of the things that staff told us makes it harder to manage agitation and have included some of the ideas about what can make it easier.

What agitated behaviours did staff experience?

Staff told us that they were coping with a range of agitated behaviours, the most common ones were verbal and physical aggression, like hitting or pinching (especially during personal care), screaming and getting upset, and repetitive behaviours like wandering up and down or repeating words.

Many staff members told us that these behaviours were often quite extreme, could get worse very quickly, were hard to predict and could last a long time. Staff also told us that agitation was different for different each person and 'no two residents are the same'.

How did agitation make staff feel?

Many staff said it could be difficult when residents become agitated, especially if it involves more than one resident. Staff sometimes felt frightened, especially when residents were aggressive. When nothing seemed to help, staff felt hopeless and like they did not have power to make things better. Some staff talked about how even though they tried to stay calm and not react badly, sometimes they felt angry or upset when residents were very agitated.

What did staff feel caused agitation?

Staff described how although it can sometimes feel like a 'guessing game' when trying to work out why someone is agitated, there were often a number of possible reasons for what may be triggering agitation. These included:

- Agitation as a sign of being upset
- Agitation as a sign of physical pain or discomfort
- Agitation as caused by how other people react
- Agitation as caused by the persons surroundings e.g. too hot, noisy, boring
- Agitation as caused by the dementia itself

For peer review only - http://bmjopen.bmj.com/site/about/guidelines.xhtml

What helps staff to prevent agitation from happening?

All the staff we spoke to said it was important to get to know the individuals in their care and build trust with the residents. There were three main things that staff said helped them to get to know residents and prevented the residents from becoming agitated. These were:

1. **Seeing the person and not the disease**

Well, I think they should be able to come in, yes, do the personal care, but while you're doing the personal care, look at the rest of the person, not only the bit you're washing and dressing, remember that they're a human being, remember that they've lived a life. (Unit manager)

2. **Connecting with previously valued identities**

Well, I always like to know what did you used to do in your time. What work do you like doing, you know. All the different things, really, in life… (Activities coordinator)

3. **Playing along rather than correcting**

While you standing arguing with someone saying, no, you're 90 and your kids are all grown up, to them they're not. They're still at school. They need help so we just say to them... so just go in their reality with them. If they're looking for their kids, the kids are at school. They're all right. (Deputy Manager)

4. **Making people feel comfortable and at home**

It's a 24 hour process and this is their home, they can get up when they like, as long as they eat and they feel comfortable, that's the most important. (Senior carer)

How do staff respond when residents are agitated?

Staff described the strategies they used when residents were agitated and how they tried to be flexible and use 'trial and error':

There's no hard and fast rules, it's just really sort of trying to read the situation really. (Care assistant)

For peer review only - http://bmjopen.bmj.com/site/about/guidelines.xhtml

Popular strategies included:

- Distracting and reassuring residents
- Giving agitated residents space
- Walking away

When these approaches did not work, staff would ask for help from senior colleagues and would sometimes get help from specialists outside of the care home. Some staff felt medication could help but said that often it had side effects and should only be used as a last resort.

What makes it harder or easier for staff to manage when residents are agitated?

1. Having the right approach makes it easier:

Staff described that being motivated, compassionate and caring, as well as recognising that you can only do your best, made it easier to do the job. They often compared this to feeling that some staff are 'only in it for the money'.

And what else gives us hope through the day? …I love my job, very much, and there is no explanation for that. You either have that or not. I could have done retail… I go through the day because I feel sorry for these people, simple as that. I… and I want to instinctively make it better. (Care assistant)

2. Communication is key:

Staff saw clear communication between team members as important. To respond well to agitation, staff needed to feel that they were not on their own and that colleagues would help if needed. Staff also said that building a good relationship with the families of residents was very important.

You do sometimes … think to yourself, I don't know if I can cope with this today but then… you take five minutes, you have a chat with one of the other carers and then we sort it out. (Care assistant)

3. Task focused vs person centred care:

Staff talked about not having enough time to spend with residents because of the staffing levels or because of all of the different jobs and tasks that they were expected to do during each shift. Staff said that they liked having 'hands on' managers who were approachable and that when they did not feel supported by management they would tend to just try and sort out problems themselves. Staff found it helpful to have good quality training but also said that learning on the job and from each other was very important.

For peer review only - http://bmjopen.bmj.com/site/about/guidelines.xhtml

I have had training [but] I've gone to management and they've taught me a different way to try and cope with it, I feel being there, dealing with it, doing it, is the best training. (Care assistant)

4. The care home industry makes it harder:

Staff talked about how television and newspapers always seem to focus on negative aspects of care, especially abuse and neglect, ignoring the good work that they saw happening and the impact of aggression from residents towards staff. This meant that families were more worried about their relatives living in a care home and staff felt this made them more worried about making mistakes or getting into trouble at work.

Sometimes it would be so lovely to hear a nice story about dementia, and staff, and what people do, and ... you don't hear things about residents lashing out at carers. (Care assistant)

Staff in all types of home talked about some of the financial problems in care homes and how they felt that focusing on business and profit made it harder for them to meet the needs of individuals.

Sometimes it can be challenging because if the budget doesn't meet ... then the staff need to be reduced, and ... the needs of the residents take second place. (Deputy Manager)

We would like to thank you again for taking part. We are also in the process of writing an article based on these findings so that a wide range of health and social care professionals will be able to learn from it.

If you have any thoughts or comments on this summary we would really appreciate your feedback. We are interested in hearing if you feel there is anything missing from this summary and whether it fits well or does not fit with your own experiences. You can either email xxx or write using the enclosed stamped addressed envelope.

For peer review only - http://bmjopen.bmj.com/site/about/guidelines.xhtml

Standards for Reporting Qualitative Research (SRQR)*

http://www.equator-network.org/reporting-guidelines/srqr/

Title and abstract

	Page/line no(s).
**Title** - Concise description of the nature and topic of the study Identifying the study as qualitative or indicating the approach (e.g., ethnography, grounded theory) or data collection methods (e.g., interview, focus group) is recommended	Page 1 line 1
**Abstract** - Summary of key elements of the study using the abstract format of the intended publication; typically includes background, purpose, methods, results, and conclusions	Page 2 line 1

Introduction

**Problem formulation** - Description and significance of the problem/phenomenon studied; review of relevant theory and empirical work; problem statement	Page 3 lines 1-28
**Purpose or research question** - Purpose of the study and specific objectives or questions	Page 3 lines 27-9

Methods

**Qualitative approach and research paradigm** - Qualitative approach (e.g., ethnography, grounded theory, case study, phenomenology, narrative research) and guiding theory if appropriate; identifying the research paradigm (e.g., postpositivist, constructivist/ interpretivist) is also recommended; rationale**	Page 4 line 19
**Researcher characteristics and reflexivity** - Researchers' characteristics that may influence the research, including personal attributes, qualifications/experience, relationship with participants, assumptions, and/or presuppositions; potential or actual interaction between researchers' characteristics and the research questions, approach, methods, results, and/or transferability	Page 1 lines 9-10<br>Page 3 lines 31-38
**Context** - Setting/site and salient contextual factors; rationale**	Page 3 lines 31-39
**Sampling strategy** - How and why research participants, documents, or events were selected; criteria for deciding when no further sampling was necessary (e.g., sampling saturation); rationale**	Page 3 lines 31-39<br>Page 4 lines 6-10
**Ethical issues pertaining to human subjects** - Documentation of approval by an appropriate ethics review board and participant consent, or explanation for lack thereof; other confidentiality and data security issues	Page 4 lines 1-12
**Data collection methods** - Types of data collected; details of data collection procedures including (as appropriate) start and stop dates of data collection and analysis, iterative process, triangulation of sources/methods, and modification of procedures in response to evolving study findings; rationale**	Page 4 lines 1-26

For peer review only

**Data collection instruments and technologies** - Description of instruments (e.g., interview guides, questionnaires) and devices (e.g., audio recorders) used for data collection; if/how the instrument(s) changed over the course of the study	Page 4 lines 4-12 Supplementary appendix 1
**Units of study** - Number and relevant characteristics of participants, documents, or events included in the study; level of participation (could be reported in results)	Page 4 lines 25-29 Table 1
**Data processing** - Methods for processing data prior to and during analysis, including transcription, data entry, data management and security, verification of data integrity, data coding, and anonymization/de-identification of excerpts	Page 4 lines 1-12
**Data analysis** - Process by which inferences, themes, etc., were identified and developed, including the researchers involved in data analysis; usually references a specific paradigm or approach; rationale**	Page 4 lines 13-22
**Techniques to enhance trustworthiness** - Techniques to enhance trustworthiness and credibility of data analysis (e.g., member checking, audit trail, triangulation); rationale**	Page 4 lines 13-22 Supplementary appendix 2

Results/findings

**Synthesis and interpretation** - Main findings (e.g., interpretations, inferences, and themes); might include development of a theory or model, or integration with prior research or theory	Page 5 line 15- page 11 line 32 Table 2
**Links to empirical data** - Evidence (e.g., quotes, field notes, text excerpts, photographs) to substantiate analytic findings	Page 5 line 15- page 11 line 32 Table 3

Discussion

**Integration with prior work, implications, transferability, and contribution(s) to the field** - Short summary of main findings; explanation of how findings and conclusions connect to, support, elaborate on, or challenge conclusions of earlier scholarship; discussion of scope of application/generalizability; identification of unique contribution(s) to scholarship in a discipline or field	Page 11 line 34 – page 12 line 29 Page 11 line 38 – page 12 line 2
**Limitations** - Trustworthiness and limitations of findings	Page 12 line 30-37

Other

**Conflicts of interest** - Potential sources of influence or perceived influence on study conduct and conclusions; how these were managed	Page 13 line 17-18
**Funding** - Sources of funding and other support; role of funders in data collection, interpretation, and reporting	Page 13 line 7-13

*The authors created the SRQR by searching the literature to identify guidelines, reporting standards, and critical appraisal criteria for qualitative research; reviewing the reference lists of retrieved sources; and contacting experts to gain feedback. The SRQR aims to improve the transparency of all aspects of qualitative research by providing clear standards for reporting qualitative research.

For peer review only - http://bmjopen.bmj.com/site/about/guidelines.xhtml

For peer review only

**The rationale should briefly discuss the justification for choosing that theory, approach, method, or technique rather than other options available, the assumptions and limitations implicit in those choices, and how those choices influence study conclusions and transferability. As appropriate, the rationale for several items might be discussed together.

Reference:

O'Brien BC, Harris IB, Beckman TJ, Reed DA, Cook DA. **Standards for reporting qualitative research: a synthesis of recommendations.** *Academic Medicine*, Vol. 89, No. 9 / Sept 2014
DOI: 10.1097/ACM.0000000000000388

For peer review only

For peer review only - http://bmjopen.bmj.com/site/about/guidelines.xhtml

BMJ Open

How do care home staff understand, manage and respond to agitation in people with dementia? A qualitative study

Journal:	*BMJ Open*
Manuscript ID	bmjopen-2018-022260.R1
Article Type:	Research
Date Submitted by the Author:	02-May-2018
Complete List of Authors:	Rapaport, Penny; University College London, Division of Psychiatry<br>Livingston, Gill; UCL, Mental Health Science<br>Hamilton, Olivia; University College London, Division of Psychiatry<br>Turner, Rebecca; University College London, Division of Psychiatry<br>Stringer, Aisling; University College London, Division of Psychiatry<br>Robertson, Sarah; University College London, Division of Psychiatry<br>Cooper, Claudia; University College London
**Primary Subject Heading**:	Geriatric medicine
Secondary Subject Heading:	Health services research, Mental health, Qualitative research
Keywords:	Dementia < NEUROLOGY, QUALITATIVE RESEARCH, Old age psychiatry < PSYCHIATRY

SCHOLARONE™
Manuscripts

How do care home staff understand, manage and respond to agitation in people with dementia? A qualitative study

Corresponding author:

Dr Penny Rapaport

Clinical Psychologist*

Tel: 0207 679 9716

Email: P.Rapaport@ucl.ac.uk

Authors:

Penny Rapaport[1,2](Mphil DclinPsy), Gill Livingston[1](MD FRCPsych), Olivia Hamilton[1](Msc), Rebecca Turner[1](MSc), Aisling Stringer[1](MSc), Sarah Robertson[1,2](PhD) and Claudia Cooper[1](PhD MRCPsych)

[1] UCL Department of Old Age Psychiatry, Division of Psychiatry, Wing A, Floor 6 Maple House, 149 Tottenham Court Rd, London, W1T 7NF, UK.

[2] North Thames CLAHRC

Word count: 4798

Tables: 1

Figures: 1

Supplementary materials:

Appendix 1: Semi structured interview schedule
Appendix 2: Summary of results for participants
Appendix 3: SRQR checklist
Appendix 4: Table of themes

For peer review only

ABSTRACT

Objectives: Little is known about how care home staff understand and respond to distress in residents living with dementia labelled as agitation. The aim of this study was to describe how care home staff understand and respond to agitation and the factors that determine how it is managed.

Design: We conducted a qualitative thematic analysis.

Setting: We recruited staff from six care homes in South East England including residential and nursing homes of differing sizes run by both the private and charity sector and located in urban and rural areas.

Participants: We interviewed 25 care home staff using purposive sampling to include staff of either sex, differing age, ethnicity, nationality and with different roles and experience.

Results: We identified four overarching themes: 1. behaviours expressing unmet need; 2. staff emotional responses to agitation; 3. understanding the individual helps, and 4. constraints on staff responses. Staff struggled with the paradox of trying to connect with the personhood of residents whilst seeing the person as separate to and, therefore, not responsible for their behaviours. Staff often felt powerless, frightened and overwhelmed and their responses were constrained by care home structures, processes and a culture of fear and scrutiny.

Conclusions: Responding to agitation expressed by residents was not a linear process and staff faced tensions and dilemmas in deciding how to respond, especially when initial strategies were unsuccessful. Future trials of psychosocial interventions should support staff to identify and respond to residents' unmet needs and include how staff can look after themselves. Interventions should build upon the approaches and strategies already being used by care home staff in routine care and address constraints.

Key words: Dementia, agitation, care homes, psychosocial interventions, care staff

ARTICLE SUMMARY

Strengths and limitations of this study

- To the best of our knowledge, this is the first study to qualitatively explore how staff in care homes understand and respond to behaviours labelled as agitation in people living with dementia.
- Understanding how staff understand and manage agitation and what makes this harder or easier has informed the development of a psychosocial intervention that reflects and addresses the complexity of delivering interventions in care home settings.
- We recruited staff based upon manager's recommendations or existing research team and staff relationships which may have resulted in recruitment bias.
- We conducted semi-structured interviews rather than directly observing care interactions therefore our analysis reflects our interpretation of staff perspectives.

For peer review only - http://bmjopen.bmj.com/site/about/guidelines.xhtml

For peer review only

INTRODUCTION

Over 70% of UK care home residents have dementia,[1] often with complex needs and high levels of behavioural and psychological symptoms of dementia (BPSD).[2] The term BPSD describes a heterogeneous range of symptoms including apathy, irritability, anxiety, depression, psychosis and agitation. As a syndrome, BPSD has been criticised as poorly defined, with authors calling for a focus on specific symptoms and targeted interventions.[3] In this study, we have therefore focused on the most common of these symptoms, agitation.[4] While agitation is variously defined, the term is often used to refer to a range of behaviours; including restlessness, pacing, repetitive vocalisations and verbally or physically aggressive behaviour.[2 5] Agitation in care homes is associated with lower quality of life and higher care costs[6-8] and is persistent and distressing.[8 9]

In a recent epidemiological care home study, we found that although severity of agitation was associated with severity of dementia this was not a linear association, with 45% of those with moderate and severe dementia experiencing clinically significant levels of agitation. From this, we concluded that the agitation cannot be fully explained in terms of worsening brain pathology,[8] reiterating the need to also conceptualise agitation in social and psychological terms. Kitwood (1997) highlights the relational nature of personhood and outlines how negative interactions between carers and people with dementia create a 'malign social psychology' undermining personhood and resulting in unmet social and psychological needs. Conversely, interventions that address these needs and promote personhood may prevent and reduce manifestations of distress such as agitation.[10]

Being labelled as agitated may increase the difficulties individuals with dementia face by impacting upon personhood; how they are perceived, understood and responded to.[11 12] This can have real consequences for people living with dementia in care homes, for example by resulting in increased use of restraint[13 14] and increased prescribing of psychotropic medications.[15] Antipsychotic medication has limited effect in reducing symptoms of agitation and leads to increased morbidity and mortality in people with dementia.[16] Other medications have limited efficacy and significant harmful side effects.[16-19] Evidence for non-pharmacological alternatives in managing agitation is mixed, with few interventions demonstrating effects after the intervention is completed.[20 21] In a systematic review of RCTs testing non-pharmacological interventions for agitation, Livingston et al (2014) found that group based activities, music therapy by protocol and sensory interventions, decrease agitation whilst the intervention is being delivered, and supervised interventions training staff in communication skills and person-centred care, reduce agitation for up to six months post-intervention.

The Needs-driven, Dementia-compromised Behaviour (NDB) theory[22] proposes that behaviours in dementia, often labelled as disruptive, arise from unmet needs. Needs may be emotional (communication, comfort or contact), recreational (stimulation and enjoyable activities) and physical (pain relief, thirst, hunger or treatment of constipation or infection). Environmental limitations can prevent needs being met, when staff are unavailable, unaware or inadequately skilled in communicating and interacting with people with dementia. Care home staff often have little training and are low paid, with high staff turnover.[23 24] Communication between staff and residents, for example, during personal care, can be dominated by instructions.[25]

For peer review only - http://bmjopen.bmj.com/site/about/guidelines.xhtml

Further understanding of the relational aspects of agitation in care homes; of how staff make sense of and respond to agitation, is necessary to facilitate development of more effective and sustainable interventions. To our knowledge, this is the first qualitative study of care home staff experiences of caring for residents with dementia experiencing agitation. We aim to describe how care home staff understand and respond to agitation and the factors that determine how it is managed. We plan to use the findings to inform the development of a sustainable and effective intervention to support staff in care homes to manage agitation.

METHODS

Setting, participants and procedure

London (Queen's Square) National Research Ethics Service (NRES) committee gave approval (reference: 14/LO/0697) for the study. We purposively selected care homes participating in MARQUE (Managing Agitation and Raising QUality of LifE in Dementia), a study involving people with dementia living in care homes[8] interviewing staff from varied care home settings: residential and nursing; differing sizes; private and charity sector; and in urban and rural areas in South East England. All of the homes we approached agreed to participate. We included staff providing direct care and support to residents with dementia, including care assistants, senior carers (who had additional responsibilities), team leaders, activities coordinators, registered nursing staff and managers. We did not interview staff in solely domestic, catering or administrative roles. We sampled purposively to ensure that we interviewed staff of either sex, differing age, ethnicity, nationality and with different roles and experience. We used a semi-structured interview schedule (see supplementary appendix 1) based on the literature, consultation with dementia family carers and research team expert opinion. Recruitment and data collection procedures are outlined in Figure 1.

Patient and public involvement

People whose lives have been affected by dementia were members of the project management group and were involved in the development of the research questions for the project. To inform the development of the interview schedule we held a focus group for family carers who had cared for a relative with agitation in a care home, discussing from their perspective, what we needed to consider in our interviews with care home staff. After completing the analysis of the data we conducted follow up focus groups presenting the findings and discussing how this may inform intervention development.

Data analysis

We took an inductive thematic analytic approach based upon the work of Braun and Clarke. [26] After completing each set of interviews (in one care home), PR listened to the recordings, reflected on initial themes and revised the interview schedule to incorporate new ideas expressed by care staff, and as part of an ongoing reflective process based upon both the emerging perspectives of the participants and the interviewer.[27] This also allowed us to check that the questions made sense to the participants, especially since over half did not speak English as a first language.

PR and one of four independent raters (OH, RT, AS, SR) systematically coded each transcript into meaningful fragments and labelled these initial codes, discrepancies were discussed and resolved.[28] PR, GL and CC then organised the data into preliminary themes, making connections between codes,

For peer review only - http://bmjopen.bmj.com/site/about/guidelines.xhtml

then displaying in matrices and diagrams developing a comprehensive picture of the phenomena in question. We discussed the coding frames within the team using the constant comparison method, [29] identifying similarities and differences in the data and refining our account in an iterative process closely grounded in the data. We ceased interviews at thematic saturation, at the point that neither of the two researchers coding an interview identified new codes and when the authors' reflections on additional interviews resulted in no further emergent themes. PR, CC and GL and OH agreed this by consensus. We sought respondent validation by sending participants summaries of the findings, allowing them to comment on the accuracy and credibility of interpretations [30] (see supplementary appendix 2). The Standards for Reporting Qualitative Research (SRQR)[31] checklist is presented in supplementary appendix 3.

RESULTS

Study participants

PR interviewed staff in six care homes in London (n=4), Kent (n=1) and Cambridge (n=1) between July 2014 and January 2015. Five homes were private and one was run by a charity. Three were nursing homes, two residential homes and one provided residential and nursing care. Table 1 summarises socio-demographic and employment characteristics of staff interviewed.

Table 1: Care staff socio-demographic and employment characteristics

Socio-demographic	Category	Care staff n (%)
Gender	Female	17 (68)
	Male	8 (32)
Ethnicity	Asian or Asian British	6 (24)
	Black or Black British	6 (24)
	White British	6 (24)
	White other	5 (20)
	Mixed other	2 (8)
English as first language	No	13 (52)
	Yes	11 (44)
	Not known	1 (4)
Staff role	Care assistant	9 (36)
	Senior carer	5 (20)
	Manager / deputy manager	5 (20)
	Team leader/unit manager	2 (8)
	Activities coordinator	2 (8)
	Registered General Nurse	2 (8)
Shift pattern	Days	18 (72)
	Days and nights	7 (28)
Length of service	Less than 1 year	4 (16)
	1 to 5 years	13 (52)
	6 to 10 years	8 (32)
Nursing qualification	Yes	10 (40)
	No	15 (60)

For peer review only - http://bmjopen.bmj.com/site/about/guidelines.xhtml

Qualitative findings

We identified four overarching themes: 1. *Behaviours expressing unmet need*; 2. *Staff emotional responses to agitation*; *3. Understanding the individual helps* and *4. Constraints on staff responses.* These are discussed below and presented in supplementary appendix 4.

1. Behaviours expressing unmet need

Staff identified a range of behaviours as expressions of unmet emotional, physical or environmental needs. Staff experienced behaviours as persistent, unpredictable and inter-related. The behaviours most commonly described were verbal and physical aggression expressed when staff provided intimate care, such as washing, dressing or assisting a resident to the toilet:

> *Like when you do the personal care he just goes with you for a while and then suddenly he'll react, it's like if you are not taking care of yourself the carers can be hit or sometimes those kinds of things, he'll just smash you like that. (Team leader; Home 1)*

1.1 Unmet physical need

Many staff described how residents were often unaware or unable to communicate that they were in pain or unwell.

> *Maybe they have pain in their legs, but they can't explain themselves. That is why I say you need patience, because they're shouting, they don't know where the pain is. (Senior carer; Home 4)*

Identifying the cause of distress involved a process of elimination and consideration of various factors such as hunger, thirst or medication. Senior staff commented that these causes may be overlooked by care staff:

> *Someone might be shouting out and instead of asking, are you in pain, or investigating why, you'll find a lot of, well, I'll do you a cup of tea. (Deputy manager; Home 2)*

1.2 Unmet emotional need

Staff drew upon knowledge of residents' past and present to understand how behaviours might arise from emotional distress. Staff conceptualised unmet emotional needs in terms of residents feeling frustrated, feeling ashamed or embarrassed during personal care and feeling insecure or abandoned. Some staff felt that physical needs were viewed as more valid or more deserving of staff responses than emotional needs, especially when working under pressure:

> *Unfortunately, what it comes to is you start to think to yourself, well, these people have real needs… because she wants the companionship, but, you know, we do have a hierarchy in terms of- is the person pain free, are they well hydrated, are they fed well, are they comfortable, and then maybe you can get to the social needs. (Deputy manager; Home 2)*

Some staff perceived overt expressions of distress such as repeated screaming or calling out as attention seeking or intentionally demanding:

> *It's a bit of a game sometimes for him I think. There's a lot of play-acting getting involved. This guy probably has lot more capacity than he thinks. (Care assistant; Home 3)*

For peer review only - http://bmjopen.bmj.com/site/about/guidelines.xhtml

1.3 Unmet environmental need

Staff described how residents may be distressed both due to a lack of interaction and under or over-stimulation:

> *Just the whole idea of them sitting in a chair is no good. I don't like that. The brain must be stimulated, even if it is a small amount. (Care assistant; Home 3)*
>
> *Like this one, who doesn't like any noise, he will stand up straightaway, like when he is having lunch, he will bang the cutlery on the table and then look at the person who is screaming. (Care assistant 2; Home 5)*

Staff also believed that care home environments contributed to unmet needs:

> *If you walk into the lounge in a care home it isn't like your home. There isn't a sofa, there's single chairs, and who has single chairs? And something that small can make a big part on someone, especially if someone is affectionate; they want to sit next to somebody. (Care assistant; Home 2)*

A number of staff described residents feeling trapped, evoking images of imprisonment:

> *He finds this home that it's a prison. (Activities coordinator; Home 3)*
>
> *I can open the door. I can have a walk outside. It's not for them. They are always going, either in this left corridor, or to the far end of the right one, or in the lounge, or in the dining area. That's it. Finished. (Care assistant; Home 4)*

2. *Staff emotional responses to agitation*

Staff sometimes struggled to respond to residents' behaviours, especially if more than one resident was involved. They reported being unable to give space to a resident or let them express discomfort, as they wanted to minimise the impact on others:

> *So it can be very difficult if he shouts all night. It's not fair on them because he's disrupting somebody and they don't sleep. Alright fair enough, he's got his got his own problems but what about the other residents. (Senior carer; Home 4)*

2.1 Feeling powerless and disheartened

Staff frequently described feeling powerless, especially when attempts to alleviate a resident's distress were not working, or if they could not make sense of a resident's behaviour. This was particularly so when residents displayed persistent, repetitive behaviours.

> *But to have someone distressed in front of you, then… someone else is getting distressed… They are looking at you to try and do something, and you can't do anything. (Deputy manager; Home 5)*

At these times staff judged themselves as not 'doing a good job' and feeling judged by others undermining their professional identity.

> *It can make you feel sometimes, when things aren't working, that you've failed… sometimes you do go home disarmed, because you feel that you haven't been able to do the best for that person. (Activities coordinator; Home 2)*

For peer review only - http://bmjopen.bmj.com/site/about/guidelines.xhtml

2.2 Feeling frightened

Staff also expressed fear of being harmed. This connected to feelings of powerlessness, especially when residents hit or shouted at staff. Anticipating harm affected how staff approached and responded to residents:

> *They are scared. It doesn't mean they don't do it, but, you know what I mean? While you're doing things, you're not doing with all the openness and things; you do it with an 'oohf'. (Care assistant; Home 4)*

Staff narrated these behaviours as 'part of the job', yet highlighted how difficult it was fearing for their own safety:

> *Sometimes it is quite traumatic to be slapped or to be kicked or to be scratched or... you know, it's not an easy thing to say, okay, I'll brush it off. (Deputy manager; Home 5)*

2.3 Trying not to react

Although staff described resident behaviours as unintentional they sometimes reacted in ways they regretted. They described trying not to react to aggression, the effort required to stay calm and how their immediate reactions could escalate behaviours.

> *It may make you react in a way that you don't want to, because you know these residents can't help their behaviours, but… you're stressed... and you may say something... you shouldn't say, or... raise your voice at a resident, which you... shouldn't do, but at that moment, you're thinking, oh, no, again. (Nurse; Home 2)*

> *I think being calm is a big thing, and not reacting because, when you're getting smacked in the face, you know, some people's natural reaction would be to say something. (Care assistant; Home 2)*

3. Understanding the individual helps

Staff found that having time to get to know and understand residents was critical to building trust and familiarity with residents. This helped them to understand and respond to residents' needs.

3.1 Seeing the person not the disease

Staff described what they termed a person-centred approach as getting to know the person with dementia informing their responses to distress behaviours. They talked about seeing residents as equals and imagining how they would feel in their situation. Staff drew upon notions of shared humanity to describe how they maintained empathy and compassion, connecting with the personhood of individuals.

> *I think they should be able to come in, yes, do the personal care, but while you're doing the personal care, look at the rest of the person, not only the bit you're washing and dressing, remember that they're a human being. (Unit manager; Home 3)*

However, staff also described behaviours that may be construed as socially unacceptable, like swearing or displaying aggression, as part of the dementia and separate to the person, moving between these different, arguably contradictory positions. This tension was apparent when staff

For peer review only - http://bmjopen.bmj.com/site/about/guidelines.xhtml

tried to talk directly to residents about behaviours considered unacceptable. Seeing people with dementia as like themselves led some to feel that they might be able to control their behaviour and were, therefore, to blame for it.

> *We said that you have to apologise to your wife because it was not nice ... swearing at her. So ... maybe he realise but he say, I don't want to, I'm not going to apologise... Maybe he just doesn't remember… when he was swearing. (Care assistant; Home 5)*

3.2 Connecting with previously valued identities

Knowing about a person's past and using this during care was viewed as a respectful way to calm residents.

> *I always like to know what did you used to do in your time. What work do you like doing, you know. All the different things really, in life. (Activities coordinator; Home 3)*

Sometimes relatives facilitated this process by sharing information and explaining 'what works'. Staff often perceived behaviours as expressing distress at losing independence and spoke of supporting individuals to reconnect with what was important to them.

> *[It] is a way of showing your independence… So giving her the money that she can pay with, she feels that she's paid... and that... she's worthy to have that sort of thing. (Activities coordinator; Home 2)*

3.3 Playing along rather than correcting

Staff struggled to know how to respond when residents were disoriented, especially when residents were unaware of the extent of their dependency. Staff at all levels talked about "playing along" or entering their reality as better than trying to orient people.

> *They may not be in the here and now, but let's go back to where they are, it's very interesting when you go back to where they are…if they feel they're a teenage girl, well, okay, we talk like a teenage girl. (Care assistant; Home 2)*

This was not simple and did not always have a positive effect. Staff found it hard to decide when to stop "playing along". They felt uncomfortable lying to residents as it could increase confusion.

> *While you standing arguing with someone saying, no, you're 90 and your kids are all grown up. To them they're … still at school...You wouldn't go as far as saying, oh, they've just gone to the shop. They'll be back in a minute because then that minute they could still be, well, where is she? (Deputy manager; Home 6)*

3.4 Making people feel comfortable and at home

Making people feel at home involved creating a stimulating and comforting environment. Staff described trying to comfort residents, particularly more impaired residents, using music, touch and other sensory stimulation, in addition to activities led by specialist staff.

> *It's a 24 hour process and this is their home, they can get up when they like, as long as they eat and they feel comfortable, that's the most important. (Senior carer; Home 5)*

For peer review only - http://bmjopen.bmj.com/site/about/guidelines.xhtml

They talked about how touch made a big difference to residents, otherwise only touched during personal care.

> *I'll say to him, do you want to dance? Because he liked to dance. He'll take me really close and we'll have a little bit of a dance. (Care assistant; Home 2)*

4. Constraints on staff responses

4.1 Procedural constraints

As noted above, getting to know the residents and delivering person-centred care and was a preferred approach, however, many acknowledged that care delivery was divided into a series of tasks, with an inherent tension between task-focused and person-focused approaches.

> *Changing in that [person-centred] direction is very difficult, because people start thinking, oh, if I do that, I might get told off. If I do that, then I won't be able to fill in the dishwasher by quarter past 11, or if I do this instead of that, then they're going to tell me off because I didn't take the bin, so it's all this kind of balancing act. (Deputy manager; Home 5)*

Additionally, there seemed to be an implicit hierarchy of how staff should respond to resident's needs, prioritising basic needs over a need for company or interaction. Staff related this to feeling that they did not have the time to engage residents in activities, relying on activities coordinators for this:

> *And you might be doing an activity with someone, the guy in one of the rooms pressed the emergency…he is almost like needs one to one care so you might be rushed off to attend to him really. It really is, the activities really does demand an extra carer I think. (Care assistant; Home 3)*

4.2 Structural constraints

Staff in all homes commented on financial challenges facing the sector, describing a business culture incompatible with delivering personalised care, particularly when it reduced staffing levels and therefore time. Staff also spoke about how, in a home that was part of a larger company, they felt anonymous and disconnected from the wider organisation. One staff member recalled having her glasses broken by a resident and the company refusing to pay for repair. Other staff said that minimum wage pay, anti-social shift patterns and staffing levels make it harder to maintain compassion:

> *Sometimes it can be challenging because if the budget doesn't meet... then the staff need to be reduced, and... The needs of the residents take second place. (Deputy manager; Home 5)*

> *And, I think, with these big homes where there are 109, 110 beds, it's too much... It is just a conveyor belt; who's next, who's next, who's next? (Deputy manager; Home 6)*

4.3 Support and training

Staff described feeling devalued by managers and not heard or taken seriously when they raised concerns. Where they felt unappreciated by residents and relatives, appreciation by managers took on additional importance.

For peer review only - http://bmjopen.bmj.com/site/about/guidelines.xhtml

You don't always feel valued for the job that you're doing; it is a very difficult job. It does have an effect... on your working practise… caring for people all day and it doesn't always feel as though staff are really cared for. (Unit manager; Home 3)

In most homes staff described how they would "keep it to ourselves", seeking support from their immediate team, assuming that managers would be unhelpful. This response was perhaps heightened by feeling that managers cannot understand their experiences as front-line carers and will not provide solutions:

Well, even if I told somebody, I don't know what they could do. What could they do? (Care assistant 2; Home 3)

Although many care staff spoke of a lack of managerial support, they (and the managers) also highlighted examples of good practice. Hands-on managers and feeling that managers had done or the job themselves left staff feeling that managers could understand their difficulties. Staff also highlighted how learning from peers and seniors through discussion and joint working enabled them to find new ways to respond.

I have had training [but] I've gone to management and they've taught me a different way to try and cope with it, I feel being there, dealing with it, doing it, is the best training. (Care assistant; Home 2)

4.4 Culture of fear and scrutiny

Staff felt that the media focussed on negative aspects of care, particularly abuse and neglect. They thought media overlooked the good practice that they saw, as well as the impact, particularly of behaviours that in other contexts would be construed as abusive, from residents towards staff, eroding staff morale.

Sometimes it would be so lovely to hear a nice story about dementia, and staff, and what people do, and... You don't hear things about residents lashing out at carers. (Care assistant; Home 2)

In some cases, negative perceptions of care homes cultivated a fear of making mistakes or getting into trouble. This stifled more creative and flexible approaches to meeting resident's unmet needs. Staff felt that appearances were sometimes prioritised over minimising distress, for example, insisting a resident changed a dirty top or came out of their room.

There's the cover your back kind of fear to people… I think that translates back into the negative thing where you don't want to try a new thing in case it hurts someone or it puts them at risk (Deputy manager; Home 2)

And they'll say, why is my mum being in bed? And, you know… obviously we tried our best and… it does annoy. We're always writing it down and just inform the Nurse so we don't get in trouble (Care assistant 2; Home 4)

DISCUSSION

Main findings

To our knowledge, this is the first qualitative study to explore how care home staff experience, understand and respond to behaviours labelled as agitation in people with dementia and what helps

For peer review only - http://bmjopen.bmj.com/site/about/guidelines.xhtml

or hinders their responses. Our aims were to describe staff experiences *and* to offer an explanatory framework to inform the development of a non-pharmacological intervention aimed at supporting staff in care homes to manage agitation. Staff described behaviours as expressions of unmet needs and wanted to deliver person-centred care, but struggled to do this when feeling overwhelmed, unsupported by management, and unsafe or fearful. They faced tensions in deciding how far to go along with a resident's disorientation or how to separate a person from their behaviour without undermining personhood. Although staff knew their response could alleviate distress they were not always able to do so. Consequently, they felt powerless and frightened, impacting upon their capacity to respond. These findings are consistent with an existing study that found behaviours perceived as aggressive, uncooperative and unpredictable were most difficult to manage.[32] When trying not to react unhelpfully and meet resident unmet needs, staff were balancing their own safety, the needs and safety of other residents and those of the resident.

The findings are consistent with the NDB theory. [22] A potential benefit of this approach, is that even if staff engage in a process of trial and error and do not fully understand what is causing a particular behaviour, this process of sense making encourages them to take a curious and person-centred position in relation to those they are caring for. Perhaps what is absent from the staff experiences is the opportunity to reflect systematically upon what is happening, as they are so immersed in it. This may explain why approaches to managing agitation that focus on communication skills [33], structured reflection [34] and delivering individualised person-centred care [35] have so far been most effective.

The impact of structural and procedural factors upon staff wellbeing and care practices has previously been documented qualitatively[36-39] and quantitatively.[24 40] Consistent with this, staff here indicated that they internalised a culture of scrutiny and fear from within and outside of care homes. This sometimes prevented staff from trying new approaches and staff felt that the care home sector was increasingly incompatible with an individualised approach. This is concerning given that inappropriate treatment of people with dementia in residential care often occurs when staff feel unable to meet clients' needs,[41] possibly because it results in emotional distancing in the context of more institutionalised care. This fits with our recently published cross-sectional survey on abuse in care homes, where abusive/neglectful behaviour was more common in homes where staff experienced more burnout and feelings of depersonalisation towards people with dementia. [42]

Clinical implications

We have built our findings into the MARQUE intervention, and are currently testing it in a randomised controlled trial. Although the term agitation is widely used in clinical and academic settings, it represents a "thin description"[43] whereas staff described a diverse range of behaviours that were broader than the definition of agitation. This reflects the rich complexity of how they experience and interpret these behaviours in care homes. Therefore, the intervention conceptualises behaviours as expressions of unmet needs in residents and introduces heuristics and practical activities, focused upon staff developing in-depth understanding of resident distress. Many strategies highlighted as useful in this study have been incorporated, such as: using tools to get to know residents better; offering comfort during routine care; allowing time for staff to share ideas and find ways to manage their own feelings. External barriers have been addressed by delivering the intervention to all staff, involving managers, developing action plans, encouraging staff to use and reflect upon new approaches, offering ongoing supervision, and leaving materials for new staff.

For peer review only - http://bmjopen.bmj.com/site/about/guidelines.xhtml

For peer review only

Limitations

Although our sampling meant that we accessed a breadth of viewpoints, contributing to the richness of this account, we directly approached staff based upon manager's recommendations or existing research team and staff relationships. There may therefore be an inherent recruitment bias. Staff could have felt pressured into taking part, which is why PR spent time before taking consent reiterating the voluntary nature of participation, answering any questions about the process, giving staff the opportunity to change their mind. In relying on interviews with care home staff, we present their narration of their experiences and perceptions of how they deliver care. We must be cautious not to overgeneralise our findings.

Future research

This study highlights the complex interrelationship between notions of personhood and needs-driven behaviours labelled as agitation[11] building upon research highlighting high skill levels required by staff expected to deliver person centred care in care homes, and the complexity of achieving this.[37] Future research should explore the long-term impact of interventions on care home culture and if and how they become embedded in care practices.

For peer review only - http://bmjopen.bmj.com/site/about/guidelines.xhtml

Author's contributions

All authors made a substantial contribution to this work. PR, CC and GL all contributed to the conception and design of the study and PR drafted the paper. All authors critically revised it and gave final approval for this version to be published. PR collected all the data and coded all the interview transcripts. OH, RT, AS, SR all coded some of the interview transcripts. PR, CC and GL then organised the data into preliminary themes.

Funding

This study was funded by a grant from the UK Economic and Social Research Council and the National Institute of Health Research Grant number NIHR/ESRC ES/L001780/1. Drs Cooper and Livingston are supported by the UCLH NIHR Biomedical Research Centre. This research was supported by the National Institute for Health Research (NIHR) Collaboration for Leadership in Applied Health Research and Care North Thames at Bart's Health NHS Trust (NIHR CLAHRC North Thames).

The views expressed are those of the author(s) and not necessarily those of the NHS, the NIHR or the Department of Health. The funders had no role in study design, data collection and analysis, decision to publish, or preparation of the manuscript.

Competing interests

The authors declare that they have no competing interests.

Data Sharing statement

The data will be made available by the authors on request.

Acknowledgments

We wish to thank all the care home staff who participated in this study and all the Alzheimer's Society research network volunteers, whose lives have been affected by dementia, for their contribution. Thank you to Joanna Murray for her contribution to the qualitative analysis.

For peer review only

For peer review only - http://bmjopen.bmj.com/site/about/guidelines.xhtml

REFERENCES

1. Prince M, Knapp M, Guerchet M, et al. Dementia UK: update. Alzheimer's Society, London, 2014.
2. Cohen-Mansfield J. Conceptualization of agitation: results based on the Cohen-Mansfield agitation inventory and the agitation behavior mapping instrument. *International Psychogeriatrics* 1997;8(S3):309-15.
3. Selbaek G. Behavioural and psychological symptoms in dementia. *Tidsskrift for den Norske laegeforening: tidsskrift for praktisk medicin, ny raekke* 2005;125(11):1500-02.
4. Margallo-Lana M, Swann A, O'Brien J, et al. Prevalence and pharmacological management of behavioural and psychological symptoms amongst dementia sufferers living in care environments. *Int J Geriatr Psychiatry* 2001;16(1):39-44.
5. Cummings J, Mintzer J, Brodaty H, et al. Agitation in cognitive disorders: International Psychogeriatric Association provisional consensus clinical and research definition. *International psychogeriatrics / IPA* 2015;27(1):7-17. doi: 10.1017/S1041610214001963
6. Morris S, Patel N, Baio G, et al. Monetary costs of agitation in older adults with Alzheimer's disease in the UK: prospective cohort study. *BMJ open* 2015;5(3):e007382. doi: 10.1136/bmjopen-2014-007382
7. Okura T, Plassman BL, Steffens DC, et al. Prevalence of Neuropsychiatric Symptoms and Their Association with Functional Limitations in Older Adults in the United States: The Aging, Demographics, and Memory Study. *Journal of the American Geriatrics Society* 2010;58(2):330-37. doi: 10.1111/j.1532-5415.2009.02680.x
8. Livingston G, Barber J, Marston L, et al. Prevalence of and associations with agitation in residents with dementia living in care homes: MARQUE cross-sectional study. *BJPsych Open* 2017;3(4):171-78. doi: 10.1192/bjpo.bp.117.005181
9. Savva GM, Zaccai J, Matthews FE, et al. Prevalence, correlates and course of behavioural and psychological symptoms of dementia in the population. *Br J Psychiatry* 2009;194(3):212-9. doi: 10.1192/bjp.bp.108.049619
10. Kitwood TM. Dementia reconsidered: The person comes first: Open university press 1997.
11. Higgs P, Gilleard C. Interrogating personhood and dementia. *Aging Ment Health* 2016;20(8):773-80. doi: 10.1080/13607863.2015.1118012
12. Kitwood T, Bredin K. Towards a theory of dementia care: personhood and well-being. *Ageing Soc* 1992;12(03):269-87. doi: 10.1017/S0144686X0000502X
13. Hantikainen V. Nursing staff perceptions of the behaviour of older nursing home residents and decision making on restraint use: a qualitative and interpretative study. *Journal of Clinical Nursing* 2001;10(2):246-56.
14. Evans D, FitzGerald M. Reasons for physically restraining patients and residents: a systematic review and content analysis. *International Journal of Nursing Studies* 2002;39(7):735-43.
15. Department of Health. The use of antipsychotic medication for people with dementia: Time for action. A report for the Minister of State for Care Services by Professor Sube Banerjee. , 2009.
16. Fox C, Crugel M, Maidment I, et al. Efficacy of memantine for agitation in Alzheimer's dementia: a randomised double-blind placebo controlled trial. *PLoS One* 2012;7(5):e35185. doi: 10.1371/journal.pone.0035185
17. Howard RJ, Juszczak E, Ballard C, et al. Donepezil for the treatment of agitation in Alzheimer's disease. *N Engl J Med* 2007;357(14):1382-92. doi: 10.1056/NEJMoa066583
18. Husebo BS, Ballard C, Sandvik R, et al. Efficacy of treating pain to reduce behavioural disturbances in residents of nursing homes with dementia: cluster randomised clinical trial. *BMJ* 2011;343:d4065. doi: 10.1136/bmj.d4065
19. Porsteinsson AP, Drye LT, Pollock BG, et al. Effect of citalopram on agitation in Alzheimer disease: the CitAD randomized clinical trial. *JAMA* 2014;311(7):682-91. doi: 10.1001/jama.2014.93

For peer review only - http://bmjopen.bmj.com/site/about/guidelines.xhtml

20. Livingston G, Kelly L, Lewis-Holmes E, et al. Non-pharmacological interventions for agitation in dementia: systematic review of randomised controlled trials. *Br J Psychiatry* 2014;205(6):436-42. doi: 10.1192/bjp.bp.113.141119
21. Jutkowitz E, Brasure M, Fuchs E, et al. Care-Delivery Interventions to Manage Agitation and Aggression in Dementia Nursing Home and Assisted Living Residents: A Systematic Review and Meta-analysis. *J Am Geriatr Soc* 2016;64(3):477-88. doi: 10.1111/jgs.13936
22. Algase DL, Beck C, Kolanowski A, et al. Need-driven dementia-compromised behavior: An alternative view of disruptive behavior. *American Journal of Alzheimer's Disease* 1996;11(6):10-19. doi: 10.1177/153331759601100603
23. Franklin B. The future care workforce: London: ILC-UK, 2014.
24. Testad I, Mikkelsen A, Ballard C, et al. Health and well-being in care staff and their relations to organizational and psychosocial factors, care staff and resident factors in nursing homes. *International journal of geriatric psychiatry* 2010;25(8):789-97. doi: 10.1002/gps.2419
25. Cohen-Mansfield J, Creedon MA, Malone T, et al. Dressing of cognitively impaired nursing home residents: Description and analysis. *The Gerontologist* 2006;46(1):89-96.
26. Braun V, Clarke V. Using thematic analysis in psychology. *Qualitative Research in Psychology* 2006;3(2):77-101. doi: 10.1191/1478088706qp063oa
27. Agee J. Developing qualitative research questions: a reflective process. *International Journal of Qualitative Studies in Education* 2009;22(4):431-47. doi: 10.1080/09518390902736512
28. Barbour RS. Checklists for improving rigour in qualitative research: a case of the tail wagging the dog? *BMJ* 2001;322(7294):1115-7. doi: 10.1136/bmj.322.7294.1115
29. Glaser BG. The Constant Comparative Method of Qualitative-Analysis. *Social Problems* 1965;12(4):436-45. doi: DOI 10.1525/sp.1965.12.4.03a00070
30. Mays N, Pope C. Qualitative research in health care. Assessing quality in qualitative research. *BMJ* 2000;320(7226):50-2. doi: 10.1136/bmj.320.7226.50
31. O'Brien BC, Harris IB, Beckman TJ, et al. Standards for Reporting Qualitative Research: A Synthesis of Recommendations. *Academic Medicine* 2014;89(9):1245-51. doi: 10.1097/Acm.0000000000000388
32. Brodaty H, Draper B, Low LF. Nursing home staff attitudes towards residents with dementia: strain and satisfaction with work. *J Adv Nurs* 2003;44(6):583-90. doi: 10.1046/j.0309-2402.2003.02848.x
33. McCallion P, Toseland RW, Lacey D, et al. Educating nursing assistants to communicate more effectively with nursing home residents with dementia. *Gerontologist* 1999;39(5):546-58. doi: 10.1093/geront/39.5.546
34. Lichtwarck B, Selbaek G, Kirkevold O, et al. Targeted Interdisciplinary Model for Evaluation and Treatment of Neuropsychiatric Symptoms: A Cluster Randomized Controlled Trial. *The American journal of geriatric psychiatry : official journal of the American Association for Geriatric Psychiatry* 2018;26(1):25-38. doi: 10.1016/j.jagp.2017.05.015
35. Chenoweth L, King MT, Jeon YH, et al. Caring for Aged Dementia Care Resident Study (CADRES) of person-centred care, dementia-care mapping, and usual care in dementia: a cluster-randomised trial. *The Lancet Neurology* 2009;8(4):317-25. doi: 10.1016/S1474-4422(09)70045-6
36. Killett A, Hyde P, Burns D, et al. How organizational factors interact to influence the quality of care of older people in the care home sector. *Journal of Health Services Research & Policy* 2013;18(1_suppl):14-22. doi: 10.1177/1355819613476016
37. Stanyon MR, Griffiths A, Thomas SA, et al. The facilitators of communication with people with dementia in a care setting: an interview study with healthcare workers. *Age Ageing* 2016;45(1):164-70. doi: 10.1093/ageing/afv161
38. Lawrence V, Fossey J, Ballard C, et al. Helping staff to implement psychosocial interventions in care homes: augmenting existing practices and meeting needs for support. *Int J Geriatr Psychiatry* 2016;31(3):284-93. doi: 10.1002/gps.4322

For peer review only - http://bmjopen.bmj.com/site/about/guidelines.xhtml

For peer review only

39. Backhouse T, Killett A, Penhale B, et al. The use of non-pharmacological interventions for dementia behaviours in care homes: findings from four in-depth, ethnographic case studies. *Age Ageing* 2016;45(6):856-63. doi: 10.1093/ageing/afw136
40. Vernooij-Dasssen MJ, Faber MJ, Olde Rikkert MG, et al. Dementia care and labour market: the role of job satisfaction. *Aging Ment Health* 2009;13(3):383-90. doi: 10.1080/13607860902861043
41. Sormunen S, Topo P, Eloniemi-Sulkava U, et al. Inappropriate treatment of people with dementia in residential and day care. *Aging Ment Health* 2007;11(3):246-55. doi: 10.1080/13607860600963539
42. Cooper C, Marston L, Barber J, et al. Do care homes deliver person-centred care? A cross-sectional survey of staff-reported abusive and positive behaviours towards residents from the MARQUE (Managing Agitation and Raising Quality of Life) English national care home survey. *PLOS ONE* 2018;13(3):e0193399. doi: 10.1371/journal.pone.0193399
43. Geertz C. Thick Description: Towards an Interpretative Theory of Culture. The Interpretation of Cultures: Selected Essays. New York: Basic Books 1973:3-30.

Legends for figures

Figure 1: Outline of recruitment and data collection procedures

For peer review only

For peer review only - http://bmjopen.bmj.com/site/about/guidelines.xhtml

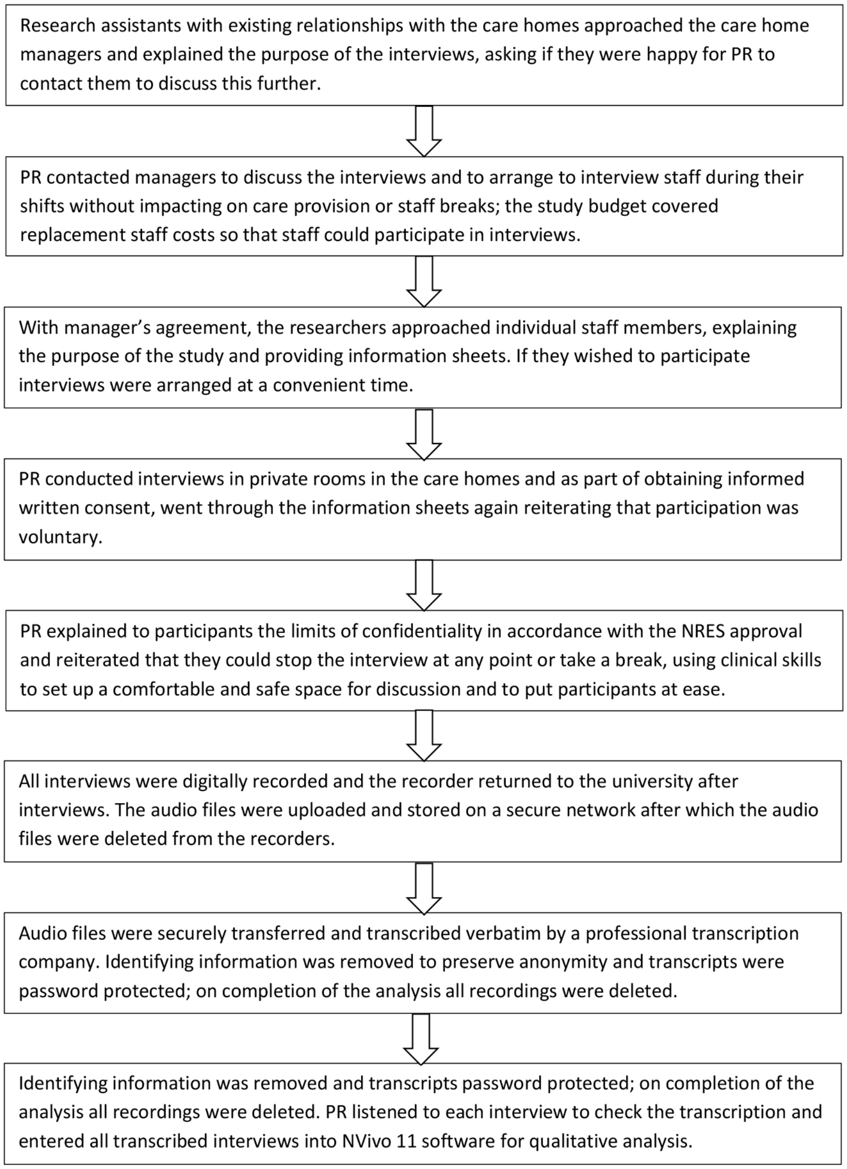

Figure 1: Outline of recruitment and data collection procedures

166x226mm (300 x 300 DPI)

UCL DIVISION OF PSYCHIATRY
FACULTY OF BRAIN SCIENCES
BLOOMSBURY CAMPUS

Study to improve agitation and quality of life for people with dementia in care homes: Care worker semi-structured interview schedule

Introductions: As you know *I am a researcher from University College London and I will be conducting and recording this interview. Everything you say is confidential but I would like you to introduce yourselves for the recording so that the typist can identify you. My name is….*

Description of the research: *Thank you for agreeing to take part in this interview, I am interested in how people working in care homes think about and cope with residents with agitation; when talking about agitation we are referring to behaviours such as restlessness, pacing, shouting and verbal or physical aggression. We are asking because we know that this is something you and your colleagues manage a lot and we want to make use of your expertise. I will be asking about your experiences of working with people with agitation; about what works and what does not and what helps or hinders.*

We will use the information to help us to develop an intervention to reduce agitation in people with dementia living in care homes and we are interested in how to make the intervention something which is practical and fits with day to day work in busy care homes.

Confidentiality: *This interview is going to be audio recorded; whatever you tell me will be anonymised for the purposes of the study so you or any other individuals you mention will not be identifiable. However if you do disclose any information that you or someone else is being harmed I will ask your permission to disclose the information to my supervisor. We respect confidentiality but cannot keep it a secret if anyone is being seriously harmed.*

If at any point during the interview you feel that you need to stop or leave the room please do tell me.

Experiences of agitation:
I want you to think of a resident you know well who has been agitated. You don't need to tell me their name.

Q. Can you tell me a bit about the person and describe their behaviour and what happened?
- *What do you think may have caused their agitation?*
- *What do you think it means when they behave in this way?*
- *What else have you noticed in other residents when they become agitated?*

(Prompt: If there is nothing much to say about this resident ask them to think of someone else)

Managing agitation:
Q. When you think about the person and situation you were just describing, how did you manage the situation?
- *What was tried?*
- *What worked well?*
- *What did not work well?*
- *What did you feel able to do at the time?*
- *What did you notice other people doing?*
- *Tell me about good ways of communication with people like this?*
- *How do decisions get made about what to do?*
- *What else have you seen work well in other situations?*

For peer review only - http://bmjopen.bmj.com/site/about/guidelines.xhtml

- *How do you think _________________ would make a difference (pain control, talking, sensory stimulation, music, activities, more communication)*
- *What would stop them working? What would make them work?*

Barriers and facilitators

Q. What makes it easier for you to manage agitation in residents?

- *What is it about you?*
- *What is it about your role?*
- *What is it about the residents / their families?*
- *What is it about your team?*
- *What is it about your managers?*
- *What is it about your organisation / employer?*
- *What is it about the media e.g. TV and newspapers?*

Q. What makes it harder for you to manage agitation in residents?

- *What is it about you?*
- *What is it about your role?*
- *What is it about the residents / their families?*
- *What is it about your team?*
- *What is it about your managers?*
- *What is it about your organisation / employer?*
- *What is it about the media e.g. TV and newspapers?*

Impact of agitation

Q. What impact does resident's agitated behaviour have upon staff?

- *What effect does it have on how you feel at work?*
- *What effect does it have upon what you feel able to do?*
- *How does it affect your team?*

Support

Q. When it is difficult, do staff get support?

- *Do you feel you can ask for help when you need it?*
- *What sort of support do you find helpful? Who from?*
- *How do you and your colleagues support each other?*
- *What gets in the way of asking for support?*

Training

Q. What training have you had to help you to manage when residents become agitated?

(If they can think of training):

- *What was most useful?*
- *What was least useful?*

If they cannot think of past training):

Q. What training do you think would help you to feel more able to manage agitation?

- *What do you think would help you to put what you learn into practice?*
- *What would make it harder to put what you learn into practice?*

(Prompt re self, role, team, managerial, organisational factors)

Other

Q. Before we finish, is there anything else you would like to mention that we have not already covered?

Would you be willing to give us feed back about any materials we may develop in future?

Thank you for taking part today.

For peer review only - http://bmjopen.bmj.com/site/about/guidelines.xhtml

What works in practice? How do care home staff understand and manage agitation in people with dementia

We are writing to you because you were interviewed by us some time ago about your experiences of caring for residents with dementia who experience agitation. We wanted to thank you again for taking part in our study and also to send you a summary of what we found out.

We interviewed 25 members of staff in six different care homes about what happens when residents become agitated, how you as staff manage and respond, and what makes it harder or easier to manage.

We have used what you and the other staff we interviewed told us about what works to develop a staff training intervention which we are currently testing in ten care homes, to see if it helps staff when residents become agitated. We have tried to address some of the things that staff told us makes it harder to manage agitation and have included some of the ideas about what can make it easier.

What agitated behaviours did staff experience?

Staff told us that they were coping with a range of agitated behaviours, the most common ones were verbal and physical aggression, like hitting or pinching (especially during personal care), screaming and getting upset, and repetitive behaviours like wandering up and down or repeating words.

Many staff members told us that these behaviours were often quite extreme, could get worse very quickly, were hard to predict and could last a long time. Staff also told us that agitation was different for different each person and 'no two residents are the same'.

How did agitation make staff feel?

Many staff said it could be difficult when residents become agitated, especially if it involves more than one resident. Staff sometimes felt frightened, especially when residents were aggressive. When nothing seemed to help, staff felt hopeless and like they did not have power to make things better. Some staff talked about how even though they tried to stay calm and not react badly, sometimes they felt angry or upset when residents were very agitated.

What did staff feel caused agitation?

Staff described how although it can sometimes feel like a 'guessing game' when trying to work out why someone is agitated, there were often a number of possible reasons for what may be triggering agitation. These included:

- Agitation as a sign of being upset
- Agitation as a sign of physical pain or discomfort
- Agitation as caused by how other people react
- Agitation as caused by the persons surroundings e.g. too hot, noisy, boring
- Agitation as caused by the dementia itself

For peer review only - http://bmjopen.bmj.com/site/about/guidelines.xhtml

What helps staff to prevent agitation from happening?

All the staff we spoke to said it was important to get to know the individuals in their care and build trust with the residents. There were three main things that staff said helped them to get to know residents and prevented the residents from becoming agitated. These were:

1. **Seeing the person and not the disease**

Well, I think they should be able to come in, yes, do the personal care, but while you're doing the personal care, look at the rest of the person, not only the bit you're washing and dressing, remember that they're a human being, remember that they've lived a life. (Unit manager)

2. **Connecting with previously valued identities**

Well, I always like to know what did you used to do in your time. What work do you like doing, you know. All the different things, really, in life… (Activities coordinator)

3. **Playing along rather than correcting**

While you standing arguing with someone saying, no, you're 90 and your kids are all grown up, to them they're not. They're still at school. They need help so we just say to them... so just go in their reality with them. If they're looking for their kids, the kids are at school. They're all right. (Deputy Manager)

4. **Making people feel comfortable and at home**

It's a 24 hour process and this is their home, they can get up when they like, as long as they eat and they feel comfortable, that's the most important. (Senior carer)

How do staff respond when residents are agitated?

Staff described the strategies they used when residents were agitated and how they tried to be flexible and use 'trial and error':

There's no hard and fast rules, it's just really sort of trying to read the situation really. (Care assistant)

For peer review only - http://bmjopen.bmj.com/site/about/guidelines.xhtml

Popular strategies included:

- Distracting and reassuring residents
- Giving agitated residents space
- Walking away

When these approaches did not work, staff would ask for help from senior colleagues and would sometimes get help from specialists outside of the care home. Some staff felt medication could help but said that often it had side effects and should only be used as a last resort.

What makes it harder or easier for staff to manage when residents are agitated?

1. Having the right approach makes it easier:

Staff described that being motivated, compassionate and caring, as well as recognising that you can only do your best, made it easier to do the job. They often compared this to feeling that some staff are 'only in it for the money'.

And what else gives us hope through the day? …I love my job, very much, and there is no explanation for that. You either have that or not. I could have done retail… I go through the day because I feel sorry for these people, simple as that. I… and I want to instinctively make it better. (Care assistant)

2. Communication is key:

Staff saw clear communication between team members as important. To respond well to agitation, staff needed to feel that they were not on their own and that colleagues would help if needed. Staff also said that building a good relationship with the families of residents was very important.

You do sometimes … think to yourself, I don't know if I can cope with this today but then… you take five minutes, you have a chat with one of the other carers and then we sort it out. (Care assistant)

3. Task focused vs person centred care:

Staff talked about not having enough time to spend with residents because of the staffing levels or because of all of the different jobs and tasks that they were expected to do during each shift. Staff said that they liked having 'hands on' managers who were approachable and that when they did not feel supported by management they would tend to just try and sort out problems themselves. Staff found it helpful to have good quality training but also said that learning on the job and from each other was very important.

For peer review only - http://bmjopen.bmj.com/site/about/guidelines.xhtml

I have had training [but] I've gone to management and they've taught me a different way to try and cope with it, I feel being there, dealing with it, doing it, is the best training. (Care assistant)

4. The care home industry makes it harder:

Staff talked about how television and newspapers always seem to focus on negative aspects of care, especially abuse and neglect, ignoring the good work that they saw happening and the impact of aggression from residents towards staff. This meant that families were more worried about their relatives living in a care home and staff felt this made them more worried about making mistakes or getting into trouble at work.

Sometimes it would be so lovely to hear a nice story about dementia, and staff, and what people do, and ... you don't hear things about residents lashing out at carers. (Care assistant)

Staff in all types of home talked about some of the financial problems in care homes and how they felt that focusing on business and profit made it harder for them to meet the needs of individuals.

Sometimes it can be challenging because if the budget doesn't meet ... then the staff need to be reduced, and ... the needs of the residents take second place. (Deputy Manager)

We would like to thank you again for taking part. We are also in the process of writing an article based on these findings so that a wide range of health and social care professionals will be able to learn from it.

If you have any thoughts or comments on this summary we would really appreciate your feedback. We are interested in hearing if you feel there is anything missing from this summary and whether it fits well or does not fit with your own experiences. You can either email xxx or write using the enclosed stamped addressed envelope.

For peer review only - http://bmjopen.bmj.com/site/about/guidelines.xhtml

Standards for Reporting Qualitative Research (SRQR)*

http://www.equator-network.org/reporting-guidelines/srqr/

Title and abstract

	Page/line no(s).
**Title** - Concise description of the nature and topic of the study Identifying the study as qualitative or indicating the approach (e.g., ethnography, grounded theory) or data collection methods (e.g., interview, focus group) is recommended	Page 1 line 1
**Abstract** - Summary of key elements of the study using the abstract format of the intended publication; typically includes background, purpose, methods, results, and conclusions	Page 2 line 1

Introduction

**Problem formulation** - Description and significance of the problem/phenomenon studied; review of relevant theory and empirical work; problem statement	Page 3 lines 1-41<br>Page 4 lines 1-7
**Purpose or research question** - Purpose of the study and specific objectives or questions	Page 4 lines 4-7

Methods

**Qualitative approach and research paradigm** - Qualitative approach (e.g., ethnography, grounded theory, case study, phenomenology, narrative research) and guiding theory if appropriate; identifying the research paradigm (e.g., postpositivist, constructivist/ interpretivist) is also recommended; rationale**	Page 4 line 25
**Researcher characteristics and reflexivity** - Researchers' characteristics that may influence the research, including personal attributes, qualifications/experience, relationship with participants, assumptions, and/or presuppositions; potential or actual interaction between researchers' characteristics and the research questions, approach, methods, results, and/or transferability	Page 1 lines 9-10<br>Page 4 lines 25-30
**Context** - Setting/site and salient contextual factors; rationale**	Page 4 lines 9-23
**Sampling strategy** - How and why research participants, documents, or events were selected; criteria for deciding when no further sampling was necessary (e.g., sampling saturation); rationale**	Page 4 lines 9-23<br>Figure 1
**Ethical issues pertaining to human subjects** - Documentation of approval by an appropriate ethics review board and participant consent, or explanation for lack thereof; other confidentiality and data security issues	Page 4 lines 10-11<br>Figure 1
**Data collection methods** - Types of data collected; details of data collection procedures including (as appropriate) start and stop dates of data collection and analysis, iterative process, triangulation of sources/methods, and modification of procedures in response to evolving study findings; rationale**	Page 4 lines 9-<br>page 5 line 2<br>Figure 1

For peer review only - http://bmjopen.bmj.com/site/about/guidelines.xhtml

**Data collection instruments and technologies** - Description of instruments (e.g., interview guides, questionnaires) and devices (e.g., audio recorders) used for data collection; if/how the instrument(s) changed over the course of the study	Page 4 lines 9-page 5 line 2 Figure 1 Supplementary appendix 1
**Units of study** - Number and relevant characteristics of participants, documents, or events included in the study; level of participation (could be reported in results)	Page 5 lines 4-8 Table 1
**Data processing** - Methods for processing data prior to and during analysis, including transcription, data entry, data management and security, verification of data integrity, data coding, and anonymization/de-identification of excerpts	Figure 1 Page 4 lines 24 – page 5 line 2
**Data analysis** - Process by which inferences, themes, etc., were identified and developed, including the researchers involved in data analysis; usually references a specific paradigm or approach; rationale**	Page 4 lines 24 – page 5 line 2
**Techniques to enhance trustworthiness** - Techniques to enhance trustworthiness and credibility of data analysis (e.g., member checking, audit trail, triangulation); rationale**	Page 4 lines 24 – page 5 line 2 Supplementary appendix 2

Results/findings

**Synthesis and interpretation** - Main findings (e.g., interpretations, inferences, and themes); might include development of a theory or model, or integration with prior research or theory	Page 5 line 23-page 11 line 22 Supplementary appendix 4
**Links to empirical data** - Evidence (e.g., quotes, field notes, text excerpts, photographs) to substantiate analytic findings	Page 5 line 23-page 11 line 22

Discussion

**Integration with prior work, implications, transferability, and contribution(s) to the field** - Short summary of main findings; explanation of how findings and conclusions connect to, support, elaborate on, or challenge conclusions of earlier scholarship; discussion of scope of application/generalizability; identification of unique contribution(s) to scholarship in a discipline or field	Page 11 line 24 – page 12 line 30
**Limitations** - Trustworthiness and limitations of findings	Page 12 line 31-39

Other

**Conflicts of interest** - Potential sources of influence or perceived influence on study conduct and conclusions; how these were managed	Page 14 line 17-18
**Funding** - Sources of funding and other support; role of funders in data collection, interpretation, and reporting	Page 14 line 7-16

*The authors created the SRQR by searching the literature to identify guidelines, reporting standards, and critical appraisal criteria for qualitative research; reviewing the reference lists of retrieved sources; and contacting experts to gain feedback. The SRQR aims to improve the transparency of all aspects of qualitative research by providing clear standards for reporting qualitative research.

For peer review only

**The rationale should briefly discuss the justification for choosing that theory, approach, method, or technique rather than other options available, the assumptions and limitations implicit in those choices, and how those choices influence study conclusions and transferability. As appropriate, the rationale for several items might be discussed together.

Reference:

O'Brien BC, Harris IB, Beckman TJ, Reed DA, Cook DA. **Standards for reporting qualitative research: a synthesis of recommendations.** *Academic Medicine*, Vol. 89, No. 9 / Sept 2014
DOI: 10.1097/ACM.0000000000000388

For peer review only

For peer review only - http://bmjopen.bmj.com/site/about/guidelines.xhtml

Appendix 4: Table of themes

Overarching themes	Sub themes
1. Behaviours expressing unmet need	1.1 Unmet physical need<br>1.2 Unmet emotional need<br>1.3 Unmet environmental need
2. Staff emotional responses to agitation	2.1 Feeling powerless and disheartened<br>2.2 Feeling frightened<br>2.3 Trying not to react
3. Understanding the individual helps	3.1 Seeing the person not the disease<br>3.2 Connecting with previously valued identities<br>3.3 Playing along with rather than correcting<br>3.4 Making people feel comfortable and at home
4. Constraints on staff responses	4.1 Procedural constraints<br>4.2 Structural constraints<br>4.3 Support and training<br>4.4 Culture of fear and scrutiny

For peer review only

BMJ Open

How do care home staff understand, manage and respond to agitation in people with dementia? A qualitative study

Journal:	*BMJ Open*
Manuscript ID	bmjopen-2018-022260.R2
Article Type:	Research
Date Submitted by the Author:	22-May-2018
Complete List of Authors:	Rapaport, Penny; University College London, Division of Psychiatry<br>Livingston, Gill; UCL, Mental Health Science<br>Hamilton, Olivia; University College London, Division of Psychiatry<br>Turner, Rebecca; University College London, Division of Psychiatry<br>Stringer, Aisling; University College London, Division of Psychiatry<br>Robertson, Sarah; University College London, Division of Psychiatry<br>Cooper, Claudia; University College London
**Primary Subject Heading**:	Geriatric medicine
Secondary Subject Heading:	Health services research, Mental health, Qualitative research
Keywords:	Dementia < NEUROLOGY, QUALITATIVE RESEARCH, Old age psychiatry < PSYCHIATRY

SCHOLARONE™
Manuscripts

How do care home staff understand, manage and respond to agitation in people with dementia? A qualitative study

Corresponding author:

Dr Penny Rapaport

Clinical Psychologist*

Tel: 0207 679 9716

Email: P.Rapaport@ucl.ac.uk

Authors:

Penny Rapaport[1,2](Mphil DclinPsy), Gill Livingston[1](MD FRCPsych), Olivia Hamilton[1](Msc), Rebecca Turner[1](MSc), Aisling Stringer[1](MSc), Sarah Robertson[1,2](PhD) and Claudia Cooper[1](PhD MRCPsych)

[1] UCL Department of Old Age Psychiatry, Division of Psychiatry, Wing A, Floor 6 Maple House, 149 Tottenham Court Rd, London, W1T 7NF, UK.

[2] North Thames CLAHRC

Word count: 4704

Tables: 1

Figures: 1

Supplementary materials:

Appendix 1: Semi structured interview schedule
Appendix 2: Summary of results for participants
Appendix 3: SRQR checklist
Appendix 4: Table of themes

For peer review only

ABSTRACT

Objectives: Little is known about how care home staff understand and respond to distress in residents living with dementia labelled as agitation. The aim of this study was to describe how care home staff understand and respond to agitation and the factors that determine how it is managed.

Design: We conducted a qualitative thematic analysis.

Setting: We recruited staff from six care homes in South East England including residential and nursing homes of differing sizes run by both the private and charity sector and located in urban and rural areas.

Participants: We interviewed 25 care home staff using purposive sampling to include staff of either sex, differing age, ethnicity, nationality and with different roles and experience.

Results: We identified four overarching themes: 1. behaviours expressing unmet need; 2. staff emotional responses to agitation; 3. understanding the individual helps, and 4. constraints on staff responses. Staff struggled with the paradox of trying to connect with the personhood of residents whilst seeing the person as separate to and, therefore, not responsible for their behaviours. Staff often felt powerless, frightened and overwhelmed and their responses were constrained by care home structures, processes and a culture of fear and scrutiny.

Conclusions: Responding to agitation expressed by residents was not a linear process and staff faced tensions and dilemmas in deciding how to respond, especially when initial strategies were unsuccessful or when attempts to respond to residents' needs were inhibited by structural and procedural constraints in the care home. Future trials of psychosocial interventions should support staff to identify and respond to residents' unmet needs and include how staff can look after themselves..

Key words: Dementia, agitation, care homes, psychosocial interventions, care staff

ARTICLE SUMMARY

Strengths and limitations of this study

- To the best of our knowledge, this is the first study to qualitatively explore how staff in care homes understand and respond to behaviours labelled as agitation in people living with dementia.
- Understanding how staff understand and manage agitation and what makes this harder or easier can inform the development of a psychosocial intervention that reflects and addresses the complexity of delivering interventions in care home settings.
- We recruited staff based upon manager's recommendations or existing research team and staff relationships which may have resulted in recruitment bias.
- We conducted semi-structured interviews rather than directly observing care interactions therefore our analysis reflects our interpretation of staff perspectives.

For peer review only - http://bmjopen.bmj.com/site/about/guidelines.xhtml

INTRODUCTION

Over 70% of UK care home residents have dementia,[1] often with complex needs and high levels of behavioural and psychological symptoms of dementia (BPSD).[2] The term BPSD describes a heterogeneous range of symptoms including apathy, irritability, anxiety, depression, psychosis and agitation. As a syndrome, BPSD has been criticised as poorly defined, with authors calling for a focus on specific symptoms and targeted interventions.[3] In this study, we have therefore focused on the most common of these symptoms, agitation.[4] While agitation is variously defined, the term is often used to refer to a range of behaviours; including restlessness, pacing, repetitive vocalisations and verbally or physically aggressive behaviour.[2 5] Agitation in care homes is associated with lower quality of life and higher care costs[6-8] and is persistent and distressing.[8 9]

In a recent epidemiological care home study, we found that although severity of agitation was associated with severity of dementia this was not a linear association, with 45% of those with moderate and severe dementia experiencing clinically significant levels of agitation. From this, we concluded that the agitation cannot be fully explained in terms of worsening brain pathology,[8] reiterating the need to also conceptualise agitation in social and psychological terms. Kitwood (1997) highlights the relational nature of personhood and outlines how negative interactions between carers and people with dementia create a ‘malign social psychology’ undermining personhood and resulting in unmet social and psychological needs. Conversely, interventions that address these needs and promote personhood may prevent and reduce manifestations of distress such as agitation.[10]

Being labelled as agitated may increase the difficulties individuals with dementia face by impacting upon personhood; how they are perceived, understood and responded to.[11 12] This can have real consequences for people living with dementia in care homes, for example by resulting in increased use of restraint[13 14] and increased prescribing of psychotropic medications.[15] Antipsychotic medication has limited effect in reducing symptoms of agitation and leads to increased morbidity and mortality in people with dementia.[16] Other medications have limited efficacy and significant harmful side effects.[16-19] Evidence for non-pharmacological alternatives in managing agitation is mixed, with few interventions demonstrating effects after the intervention is completed.[20 21]

The Needs-driven, Dementia-compromised Behaviour (NDB) theory[22] proposes that behaviours in dementia, often labelled as disruptive, arise from unmet needs. Needs may be emotional (communication, comfort or contact), recreational (stimulation and enjoyable activities) and physical (pain relief, thirst, hunger or treatment of constipation or infection). Environmental limitations can prevent needs being met, when staff are unavailable, unaware or inadequately skilled in communicating and interacting with people with dementia. Care home staff often have little training and are low paid, with high staff turnover.[23 24] Communication between staff and residents, for example, during personal care, can be dominated by instructions.[25]

Further understanding of the relational aspects of agitation in care homes; of how staff make sense of and respond to agitation, is necessary to facilitate development of more effective and sustainable interventions. To our knowledge, this is the first qualitative study of care home staff experiences of caring for residents with dementia experiencing agitation. We aim to describe how care home staff understand and respond to agitation and the factors that determine how it is managed.

For peer review only

METHODS

Setting, participants and procedure

London (Queen's Square) National Research Ethics Service (NRES) committee gave approval (reference: 14/LO/0697) for the study. We purposively selected care homes participating in MARQUE (Managing Agitation and Raising QUality of LifE in Dementia), a study involving people with dementia living in care homes[8] interviewing staff from varied care home settings: residential and nursing; differing sizes; private and charity sector; and in urban and rural areas in South East England. All of the homes we approached agreed to participate. We included staff providing direct care and support to residents with dementia, including care assistants, senior carers (who had additional responsibilities), team leaders, activities coordinators, registered nursing staff and managers. We did not interview staff in solely domestic, catering or administrative roles. We sampled purposively to ensure that we interviewed staff of either sex, differing age, ethnicity, nationality and with different roles and experience. We used a semi-structured interview schedule (see supplementary appendix 1) based on the literature, consultation with dementia family carers and research team expert opinion. Recruitment and data collection procedures are outlined in Figure 1.

Patient and public involvement

People whose lives have been affected by dementia were members of the project management group and were involved in the development of the research questions for the project. To inform the development of the interview schedule we held a focus group for family carers who had cared for a relative with agitation in a care home, discussing from their perspective, what we needed to consider in our interviews with care home staff. After completing the analysis of the data we conducted follow up focus groups presenting the findings and discussing how this may inform intervention development.

Data analysis

We took an inductive thematic analytic approach based upon the work of Braun and Clarke. [26] After completing each set of interviews (in one care home), PR listened to the recordings, reflected on initial themes and revised the interview schedule to incorporate new ideas expressed by care staff, and as part of an ongoing reflective process based upon both the emerging perspectives of the participants and the interviewer.[27] This also allowed us to check that the questions made sense to the participants, especially since over half did not speak English as a first language.

PR and one of four independent raters (OH, RT, AS, SR) systematically coded each transcript into meaningful fragments and labelled these initial codes, discrepancies were discussed and resolved.[28] PR, GL and CC then organised the data into preliminary themes, making connections between codes, then displaying in matrices and diagrams developing a comprehensive picture of the phenomena in question. We discussed the coding frames within the team using the constant comparison method, [29] identifying similarities and differences in the data and refining our account in an iterative process closely grounded in the data. We ceased interviews at thematic saturation, at the point that neither of the two researchers coding an interview identified new codes and when the authors' reflections on additional interviews resulted in no further emergent themes. PR, CC and GL and OH agreed this by consensus. We sought respondent validation by sending participants summaries of the findings, allowing them to comment on the accuracy and credibility of interpretations [30] (see supplementary

For peer review only - http://bmjopen.bmj.com/site/about/guidelines.xhtml

appendix 2). The Standards for Reporting Qualitative Research (SRQR)[31] checklist is presented in supplementary appendix 3.

RESULTS

Study participants

PR interviewed staff in six care homes in London (n=4), Kent (n=1) and Cambridge (n=1) between July 2014 and January 2015. Five homes were private and one was run by a charity. Three were nursing homes, two residential homes and one provided residential and nursing care. Table 1 summarises socio-demographic and employment characteristics of staff interviewed.

Table 1: Care staff socio-demographic and employment characteristics

Socio-demographic	Category	Care staff n (%)
Gender	Female	17 (68)
	Male	8 (32)
Ethnicity	Asian or Asian British	6 (24)
	Black or Black British	6 (24)
	White British	6 (24)
	White other	5 (20)
	Mixed other	2 (8)
English as first language	No	13 (52)
	Yes	11 (44)
	Not known	1 (4)
Staff role	Care assistant	9 (36)
	Senior carer	5 (20)
	Manager / deputy manager	5 (20)
	Team leader/unit manager	2 (8)
	Activities coordinator	2 (8)
	Registered General Nurse	2 (8)
Shift pattern	Days	18 (72)
	Days and nights	7 (28)
Length of service	Less than 1 year	4 (16)
	1 to 5 years	13 (52)
	6 to 10 years	8 (32)
Nursing qualification	Yes	10 (40)
	No	15 (60)

Qualitative findings

We identified four overarching themes: 1. *Behaviours expressing unmet need*; 2. *Staff emotional responses to agitation*; *3. Understanding the individual helps* and *4. Constraints on staff responses.* These are discussed below and presented in supplementary appendix 4.

1. Behaviours expressing unmet need

Staff identified a range of behaviours as expressions of unmet emotional, physical or environmental needs. Staff experienced behaviours as persistent, unpredictable and inter-related. The behaviours most commonly described were verbal and physical aggression expressed when staff provided intimate care, such as washing, dressing or assisting a resident to the toilet:

For peer review only - http://bmjopen.bmj.com/site/about/guidelines.xhtml

For peer review only

Like when you do the personal care he just goes with you for a while and then suddenly he'll react, it's like if you are not taking care of yourself the carers can be hit or sometimes those kinds of things, he'll just smash you like that. (Team leader; Home 1)

1.1 Unmet physical need

Many staff described how residents were often unaware or unable to communicate that they were in pain or unwell.

Maybe they have pain in their legs, but they can't explain themselves. That is why I say you need patience, because they're shouting, they don't know where the pain is. (Senior carer; Home 4)

Identifying the cause of distress involved a process of elimination and consideration of various factors such as hunger, thirst or medication. Senior staff commented that these causes may be overlooked by care staff:

Someone might be shouting out and instead of asking, are you in pain, or investigating why, you'll find a lot of, well, I'll do you a cup of tea. (Deputy manager; Home 2)

1.2 Unmet emotional need

Staff drew upon knowledge of residents' past and present to understand how behaviours might arise from emotional distress. Staff conceptualised unmet emotional needs in terms of residents feeling frustrated, feeling ashamed or embarrassed during personal care and feeling insecure or abandoned. Some staff felt that physical needs were viewed as more valid or more deserving of staff responses than emotional needs, especially when working under pressure:

Unfortunately, what it comes to is you start to think to yourself, well, these people have real needs… because she wants the companionship, but, you know, we do have a hierarchy in terms of- is the person pain free, are they well hydrated, are they fed well, are they comfortable, and then maybe you can get to the social needs. (Deputy manager; Home 2)

Some staff perceived overt expressions of distress such as repeated screaming or calling out as attention seeking or intentionally demanding:

It's a bit of a game sometimes for him I think. There's a lot of play-acting getting involved. This guy probably has lot more capacity than he thinks. (Care assistant; Home 3)

1.3 Unmet environmental need

Staff described how residents may be distressed both due to a lack of interaction and under or over-stimulation:

Just the whole idea of them sitting in a chair is no good. I don't like that. The brain must be stimulated, even if it is a small amount. (Care assistant; Home 3)

Like this one, who doesn't like any noise, he will stand up straightaway, like when he is having lunch, he will bang the cutlery on the table and then look at the person who is screaming. (Care assistant 2; Home 5)

For peer review only - http://bmjopen.bmj.com/site/about/guidelines.xhtml

For peer review only

Staff also believed that care home environments contributed to unmet needs:

> *If you walk into the lounge in a care home it isn't like your home. There isn't a sofa, there's single chairs, and who has single chairs? And something that small can make a big part on someone, especially if someone is affectionate; they want to sit next to somebody. (Care assistant; Home 2)*

A number of staff described residents feeling trapped, evoking images of imprisonment:

> *He finds this home that it's a prison. (Activities coordinator; Home 3)*

> *I can open the door. I can have a walk outside. It's not for them. They are always going, either in this left corridor, or to the far end of the right one, or in the lounge, or in the dining area. That's it. Finished. (Care assistant; Home 4)*

2. Staff emotional responses to agitation

Staff sometimes struggled to respond to residents' behaviours, especially if more than one resident was involved. They reported being unable to give space to a resident or let them express discomfort, as they wanted to minimise the impact on others:

> *So it can be very difficult if he shouts all night. It's not fair on them because he's disrupting somebody and they don't sleep. Alright fair enough, he's got his got his own problems but what about the other residents. (Senior carer; Home 4)*

2.1 Feeling powerless and disheartened

Staff frequently described feeling powerless, especially when attempts to alleviate a resident's distress were not working, or if they could not make sense of a resident's behaviour. This was particularly so when residents displayed persistent, repetitive behaviours.

> *But to have someone distressed in front of you, then… someone else is getting distressed… They are looking at you to try and do something, and you can't do anything. (Deputy manager; Home 5)*

At these times staff judged themselves as not 'doing a good job' and feeling judged by others undermining their professional identity.

> *It can make you feel sometimes, when things aren't working, that you've failed… sometimes you do go home disarmed, because you feel that you haven't been able to do the best for that person. (Activities coordinator; Home 2)*

2.2 Feeling frightened

Staff also expressed fear of being harmed. This connected to feelings of powerlessness, especially when residents hit or shouted at staff. Anticipating harm affected how staff approached and responded to residents:

> *They are scared. It doesn't mean they don't do it, but, you know what I mean? While you're doing things, you're not doing with all the openness and things; you do it with an 'oohf'. (Care assistant; Home 4)*

Staff narrated these behaviours as 'part of the job', yet highlighted how difficult it was fearing for their own safety:

For peer review only - http://bmjopen.bmj.com/site/about/guidelines.xhtml

> *Sometimes it is quite traumatic to be slapped or to be kicked or to be scratched or... you know, it's not an easy thing to say, okay, I'll brush it off. (Deputy manager; Home 5)*

2.3 Trying not to react

Although staff described resident behaviours as unintentional they sometimes reacted in ways they regretted. They described trying not to react to aggression, the effort required to stay calm and how their immediate reactions could escalate behaviours.

> *It may make you react in a way that you don't want to, because you know these residents can't help their behaviours, but… you're stressed... and you may say something... you shouldn't say, or... raise your voice at a resident, which you... shouldn't do, but at that moment, you're thinking, oh, no, again. (Nurse; Home 2)*

> *I think being calm is a big thing, and not reacting because, when you're getting smacked in the face, you know, some people's natural reaction would be to say something. (Care assistant; Home 2)*

3. Understanding the individual helps

Staff found that having time to get to know and understand residents was critical to building trust and familiarity with residents. This helped them to understand and respond to residents' needs.

3.1 Seeing the person not the disease

Staff described what they termed a person-centred approach as getting to know the person with dementia informing their responses to distress behaviours. They talked about seeing residents as equals and imagining how they would feel in their situation. Staff drew upon notions of shared humanity to describe how they maintained empathy and compassion, connecting with the personhood of individuals.

> *I think they should be able to come in, yes, do the personal care, but while you're doing the personal care, look at the rest of the person, not only the bit you're washing and dressing, remember that they're a human being. (Unit manager; Home 3)*

However, staff also described behaviours that may be construed as socially unacceptable, like swearing or displaying aggression, as part of the dementia and separate to the person, moving between these different, arguably contradictory positions. This tension was apparent when staff tried to talk directly to residents about behaviours considered unacceptable. Seeing people with dementia as like themselves led some to feel that they might be able to control their behaviour and were, therefore, to blame for it.

> *We said that you have to apologise to your wife because it was not nice ... swearing at her. So ... maybe he realise but he say, I don't want to, I'm not going to apologise... Maybe he just doesn't remember… when he was swearing. (Care assistant; Home 5)*

3.2 Connecting with previously valued identities

Knowing about a person's past and using this during care was viewed as a respectful way to calm residents.

For peer review only - http://bmjopen.bmj.com/site/about/guidelines.xhtml

I always like to know what did you used to do in your time. What work do you like doing, you know. All the different things really, in life. (Activities coordinator; Home 3)

Sometimes relatives facilitated this process by sharing information and explaining 'what works'. Staff often perceived behaviours as expressing distress at losing independence and spoke of supporting individuals to reconnect with what was important to them.

[It] is a way of showing your independence… So giving her the money that she can pay with, she feels that she's paid... and that... she's worthy to have that sort of thing. (Activities coordinator; Home 2)

3.3 Playing along rather than correcting

Staff struggled to know how to respond when residents were disoriented, especially when residents were unaware of the extent of their dependency. Staff at all levels talked about "playing along" or entering their reality as better than trying to orient people.

They may not be in the here and now, but let's go back to where they are, it's very interesting when you go back to where they are…if they feel they're a teenage girl, well, okay, we talk like a teenage girl. (Care assistant; Home 2)

This was not simple and did not always have a positive effect. Staff found it hard to decide when to stop "playing along". They felt uncomfortable lying to residents as it could increase confusion.

While you standing arguing with someone saying, no, you're 90 and your kids are all grown up. To them they're … still at school...You wouldn't go as far as saying, oh, they've just gone to the shop. They'll be back in a minute because then that minute they could still be, well, where is she? (Deputy manager; Home 6)

3.4 Making people feel comfortable and at home

Making people feel at home involved creating a stimulating and comforting environment. Staff described trying to comfort residents, particularly more impaired residents, using music, touch and other sensory stimulation, in addition to activities led by specialist staff.

It's a 24 hour process and this is their home, they can get up when they like, as long as they eat and they feel comfortable, that's the most important. (Senior carer; Home 5)

They talked about how touch made a big difference to residents, otherwise only touched during personal care.

I'll say to him, do you want to dance? Because he liked to dance. He'll take me really close and we'll have a little bit of a dance. (Care assistant; Home 2)

4. Constraints on staff responses

4.1 Procedural constraints

As noted above, getting to know the residents and delivering person-centred care and was a preferred approach, however, many acknowledged that care delivery was divided into a series of tasks, with an inherent tension between task-focused and person-focused approaches.

For peer review only - http://bmjopen.bmj.com/site/about/guidelines.xhtml

Changing in that [person-centred] direction is very difficult, because people start thinking, oh, if I do that, I might get told off. If I do that, then I won't be able to fill in the dishwasher by quarter past 11, or if I do this instead of that, then they're going to tell me off because I didn't take the bin, so it's all this kind of balancing act. (Deputy manager; Home 5)

Additionally, there seemed to be an implicit hierarchy of how staff should respond to resident's needs, prioritising basic needs over a need for company or interaction. Staff related this to feeling that they did not have the time to engage residents in activities, relying on activities coordinators for this:

And you might be doing an activity with someone, the guy in one of the rooms pressed the emergency…he is almost like needs one to one care so you might be rushed off to attend to him really. It really is, the activities really does demand an extra carer I think. (Care assistant; Home 3)

4.2 Structural constraints

Staff in all homes commented on financial challenges facing the sector, describing a business culture incompatible with delivering personalised care, particularly when it reduced staffing levels and therefore time. Staff also spoke about how, in a home that was part of a larger company, they felt anonymous and disconnected from the wider organisation. One staff member recalled having her glasses broken by a resident and the company refusing to pay for repair. Other staff said that minimum wage pay, anti-social shift patterns and staffing levels make it harder to maintain compassion:

Sometimes it can be challenging because if the budget doesn't meet... then the staff need to be reduced, and... The needs of the residents take second place. (Deputy manager; Home 5)

And, I think, with these big homes where there are 109, 110 beds, it's too much... It is just a conveyor belt; who's next, who's next, who's next? (Deputy manager; Home 6)

4.3 Support and training

Staff described feeling devalued by managers and not heard or taken seriously when they raised concerns. Where they felt unappreciated by residents and relatives, appreciation by managers took on additional importance.

You don't always feel valued for the job that you're doing; it is a very difficult job. It does have an effect... on your working practise… caring for people all day and it doesn't always feel as though staff are really cared for. (Unit manager; Home 3)

In most homes staff described how they would "keep it to ourselves", seeking support from their immediate team, assuming that managers would be unhelpful. This response was perhaps heightened by feeling that managers cannot understand their experiences as front-line carers and will not provide solutions:

Well, even if I told somebody, I don't know what they could do. What could they do? (Care assistant 2; Home 3)

Although many care staff spoke of a lack of managerial support, they (and the managers) also highlighted examples of good practice. Hands-on managers and feeling that managers had done or

For peer review only - http://bmjopen.bmj.com/site/about/guidelines.xhtml

the job themselves left staff feeling that managers could understand their difficulties. Staff also highlighted how learning from peers and seniors through discussion and joint working enabled them to find new ways to respond.

> *I have had training [but] I've gone to management and they've taught me a different way to try and cope with it, I feel being there, dealing with it, doing it, is the best training. (Care assistant; Home 2)*

4.4 Culture of fear and scrutiny

Staff felt that the media focussed on negative aspects of care, particularly abuse and neglect. They thought media overlooked the good practice that they saw, as well as the impact, particularly of behaviours that in other contexts would be construed as abusive, from residents towards staff, eroding staff morale.

> *Sometimes it would be so lovely to hear a nice story about dementia, and staff, and what people do, and... You don't hear things about residents lashing out at carers. (Care assistant; Home 2)*

In some cases, negative perceptions of care homes cultivated a fear of making mistakes or getting into trouble. This stifled more creative and flexible approaches to meeting resident's unmet needs. Staff felt that appearances were sometimes prioritised over minimising distress, for example, insisting a resident changed a dirty top or came out of their room.

> *There's the cover your back kind of fear to people… I think that translates back into the negative thing where you don't want to try a new thing in case it hurts someone or it puts them at risk (Deputy manager; Home 2)*

> *And they'll say, why is my mum being in bed? And, you know… obviously we tried our best and… it does annoy. We're always writing it down and just inform the Nurse so we don't get in trouble (Care assistant 2; Home 4)*

DISCUSSION

Main findings

To our knowledge, this is the first qualitative study to explore how care home staff experience, understand and respond to behaviours labelled as agitation in people with dementia and what helps or hinders their responses. The findings indicate that staff in care homes understand behaviours labelled as agitation as multi-faceted and relational, consistent with conclusions of the MARQUE cross sectional study that agitation is not entirely explained in terms of brain pathology.[8]

The findings support the NDB theory with staff explaining agitation as expressions of unmet needs in residents. [22] Even if staff engage in a process of trial and error and do not fully understand what is causing a particular behaviour, this process of sense making encourages them to take a curious and person-centred position in relation to those they are caring for. It may also highlight the range of behavioural responses available to them, rather than leaving staff feeling that nothing can be done; reinforcing that finding ways to address these needs by getting to know the individual can prevent or reduce agitation.[10]

Understanding the needs of residents was not straightforward for staff and although some staff felt that unmet physical needs could be viewed as more valid than emotional and social needs, they

For peer review only - http://bmjopen.bmj.com/site/about/guidelines.xhtml

were also felt to be frequently overlooked. Existing research has found that pain and discomfort is under-detected in those with severe dementia in care homes and that discomfort is associated with higher levels of verbal aggression.[32] This is important since in the presence of behaviours perceived as aggressive, staff felt powerless and frightened, impacting upon their capacity to respond to resident underlying needs. These findings are consistent with an existing study that found behaviours perceived as aggressive, uncooperative and unpredictable were felt to be most difficult to manage.[33]

Staff wanted to deliver person-centred care, but struggled to do this when feeling overwhelmed, unsupported by management, and unsafe or fearful. They faced tensions in deciding how far to go along with a resident's disorientation or how to separate a person from their behaviour without undermining personhood. In his work on personhood and dementia, Kitwood highlighted the relational dimension of personhood as connected to both 'cared for' and 'carer' [12]. Generally however, this has been related to how those caring for people with dementia can enhance or diminish personhood through their responses and ultimately this may result in staff being blamed or seen as the cause of problems by not being person-centred or doing a good enough job.

The impact of structural and procedural factors upon staff wellbeing and care practices has previously been documented qualitatively[34-37] and quantitatively.[24 38] Consistent with this, staff here indicated that they internalised a culture of scrutiny and fear from within and outside of care homes. This sometimes prevented staff from trying new approaches and staff felt that the care home sector was increasingly incompatible with an individualised approach. This is concerning given that inappropriate treatment of people with dementia in residential care often occurs when staff feel unable to meet clients' needs,[39] possibly because it results in emotional distancing in the context of more institutionalised care. This fits with our recently published cross-sectional survey on abuse in care homes, where abusive/neglectful behaviour was more common in homes where staff experienced more burnout and feelings of depersonalisation towards people with dementia.[40]

Clinical implications

These findings have implications for the development of sustainable and practical interventions which build upon approaches that staff find useful and address the practical and structural constraints discussed above. These findings reinforce the need to find ways to support staff to manage their own emotional responses and reactions when residents are agitated, as well as supporting them to reflect systematically on recognising and meeting residents' unmet needs.

Limitations

Although our sampling meant that we accessed a breadth of viewpoints, contributing to the richness of this account, we directly approached staff based upon manager's recommendations or existing research team and staff relationships. There may therefore be an inherent recruitment bias. Staff could have felt pressured into taking part, which is why PR spent time before taking consent reiterating the voluntary nature of participation, answering any questions about the process, giving staff the opportunity to change their mind. In relying on interviews with care home staff, we present their narration of their experiences and perceptions of how they deliver care. We must be cautious not to overgeneralise our findings.

For peer review only - http://bmjopen.bmj.com/site/about/guidelines.xhtml

Future research

This study highlights the complex interrelationship between notions of personhood and needs-driven behaviours labelled as agitation[11] building upon research highlighting high skill levels required by staff expected to deliver person centred care in care homes, and the complexity of achieving this.[35] Future research should explore the long-term impact of interventions designed to reduce agitation, which incorporate this complexity, on care home culture and if and how they become embedded in care practices.

For peer review only

For peer review only - http://bmjopen.bmj.com/site/about/guidelines.xhtml

Author's contributions

All authors made a substantial contribution to this work. PR, CC and GL all contributed to the conception and design of the study and PR drafted the paper. All authors critically revised it and gave final approval for this version to be published. PR collected all the data and coded all the interview transcripts. OH, RT, AS, SR all coded some of the interview transcripts. PR, CC and GL then organised the data into preliminary themes.

Funding

This study was funded by a grant from the UK Economic and Social Research Council and the National Institute of Health Research Grant number NIHR/ESRC ES/L001780/1. Drs Cooper and Livingston are supported by the UCLH NIHR Biomedical Research Centre. This research was supported by the National Institute for Health Research (NIHR) Collaboration for Leadership in Applied Health Research and Care North Thames at Bart's Health NHS Trust (NIHR CLAHRC North Thames).

The views expressed are those of the author(s) and not necessarily those of the NHS, the NIHR or the Department of Health. The funders had no role in study design, data collection and analysis, decision to publish, or preparation of the manuscript.

Competing interests

The authors declare that they have no competing interests.

Data Sharing statement

The data will be made available by the authors on request.

Acknowledgments

We wish to thank all the care home staff who participated in this study and all the Alzheimer's Society research network volunteers, whose lives have been affected by dementia, for their contribution. Thank you to Joanna Murray for her contribution to the qualitative analysis.

For peer review only

REFERENCES

1. Prince M, Knapp M, Guerchet M, et al. Dementia UK: update. Alzheimer's Society, London, 2014.
2. Cohen-Mansfield J. Conceptualization of agitation: results based on the Cohen-Mansfield agitation inventory and the agitation behavior mapping instrument. *International Psychogeriatrics* 1997;8(S3):309-15.
3. Selbaek G. Behavioural and psychological symptoms in dementia. *Tidsskrift for den Norske laegeforening: tidsskrift for praktisk medicin, ny raekke* 2005;125(11):1500-02.
4. Margallo-Lana M, Swann A, O'Brien J, et al. Prevalence and pharmacological management of behavioural and psychological symptoms amongst dementia sufferers living in care environments. *Int J Geriatr Psychiatry* 2001;16(1):39-44.
5. Cummings J, Mintzer J, Brodaty H, et al. Agitation in cognitive disorders: International Psychogeriatric Association provisional consensus clinical and research definition. *International psychogeriatrics / IPA* 2015;27(1):7-17. doi: 10.1017/S1041610214001963
6. Morris S, Patel N, Baio G, et al. Monetary costs of agitation in older adults with Alzheimer's disease in the UK: prospective cohort study. *BMJ open* 2015;5(3):e007382. doi: 10.1136/bmjopen-2014-007382
7. Okura T, Plassman BL, Steffens DC, et al. Prevalence of Neuropsychiatric Symptoms and Their Association with Functional Limitations in Older Adults in the United States: The Aging, Demographics, and Memory Study. *Journal of the American Geriatrics Society* 2010;58(2):330-37. doi: 10.1111/j.1532-5415.2009.02680.x
8. Livingston G, Barber J, Marston L, et al. Prevalence of and associations with agitation in residents with dementia living in care homes: MARQUE cross-sectional study. *BJPsych Open* 2017;3(4):171-78. doi: 10.1192/bjpo.bp.117.005181
9. Savva GM, Zaccai J, Matthews FE, et al. Prevalence, correlates and course of behavioural and psychological symptoms of dementia in the population. *Br J Psychiatry* 2009;194(3):212-9. doi: 10.1192/bjp.bp.108.049619
10. Kitwood TM. Dementia reconsidered: The person comes first: Open university press 1997.
11. Higgs P, Gilleard C. Interrogating personhood and dementia. *Aging Ment Health* 2016;20(8):773-80. doi: 10.1080/13607863.2015.1118012
12. Kitwood T, Bredin K. Towards a theory of dementia care: personhood and well-being. *Ageing Soc* 1992;12(03):269-87. doi: 10.1017/S0144686X0000502X
13. Hantikainen V. Nursing staff perceptions of the behaviour of older nursing home residents and decision making on restraint use: a qualitative and interpretative study. *Journal of Clinical Nursing* 2001;10(2):246-56.
14. Evans D, FitzGerald M. Reasons for physically restraining patients and residents: a systematic review and content analysis. *International Journal of Nursing Studies* 2002;39(7):735-43.
15. Department of Health. The use of antipsychotic medication for people with dementia: Time for action. A report for the Minister of State for Care Services by Professor Sube Banerjee. , 2009.
16. Fox C, Crugel M, Maidment I, et al. Efficacy of memantine for agitation in Alzheimer's dementia: a randomised double-blind placebo controlled trial. *PLoS One* 2012;7(5):e35185. doi: 10.1371/journal.pone.0035185
17. Howard RJ, Juszczak E, Ballard C, et al. Donepezil for the treatment of agitation in Alzheimer's disease. *N Engl J Med* 2007;357(14):1382-92. doi: 10.1056/NEJMoa066583
18. Husebo BS, Ballard C, Sandvik R, et al. Efficacy of treating pain to reduce behavioural disturbances in residents of nursing homes with dementia: cluster randomised clinical trial. *BMJ* 2011;343:d4065. doi: 10.1136/bmj.d4065
19. Porsteinsson AP, Drye LT, Pollock BG, et al. Effect of citalopram on agitation in Alzheimer disease: the CitAD randomized clinical trial. *JAMA* 2014;311(7):682-91. doi: 10.1001/jama.2014.93

For peer review only - http://bmjopen.bmj.com/site/about/guidelines.xhtml

20. Livingston G, Kelly L, Lewis-Holmes E, et al. Non-pharmacological interventions for agitation in dementia: systematic review of randomised controlled trials. *Br J Psychiatry* 2014;205(6):436-42. doi: 10.1192/bjp.bp.113.141119
21. Jutkowitz E, Brasure M, Fuchs E, et al. Care-Delivery Interventions to Manage Agitation and Aggression in Dementia Nursing Home and Assisted Living Residents: A Systematic Review and Meta-analysis. *J Am Geriatr Soc* 2016;64(3):477-88. doi: 10.1111/jgs.13936
22. Algase DL, Beck C, Kolanowski A, et al. Need-driven dementia-compromised behavior: An alternative view of disruptive behavior. *American Journal of Alzheimer's Disease* 1996;11(6):10-19. doi: 10.1177/153331759601100603
23. Franklin B. The future care workforce: London: ILC-UK, 2014.
24. Testad I, Mikkelsen A, Ballard C, et al. Health and well-being in care staff and their relations to organizational and psychosocial factors, care staff and resident factors in nursing homes. *International journal of geriatric psychiatry* 2010;25(8):789-97. doi: 10.1002/gps.2419
25. Cohen-Mansfield J, Creedon MA, Malone T, et al. Dressing of cognitively impaired nursing home residents: Description and analysis. *The Gerontologist* 2006;46(1):89-96.
26. Braun V, Clarke V. Using thematic analysis in psychology. *Qualitative Research in Psychology* 2006;3(2):77-101. doi: 10.1191/1478088706qp063oa
27. Agee J. Developing qualitative research questions: a reflective process. *International Journal of Qualitative Studies in Education* 2009;22(4):431-47. doi: 10.1080/09518390902736512
28. Barbour RS. Checklists for improving rigour in qualitative research: a case of the tail wagging the dog? *BMJ* 2001;322(7294):1115-7. doi: 10.1136/bmj.322.7294.1115
29. Glaser BG. The Constant Comparative Method of Qualitative-Analysis. *Social Problems* 1965;12(4):436-45. doi: DOI 10.1525/sp.1965.12.4.03a00070
30. Mays N, Pope C. Qualitative research in health care. Assessing quality in qualitative research. *BMJ* 2000;320(7226):50-2. doi: 10.1136/bmj.320.7226.50
31. O'Brien BC, Harris IB, Beckman TJ, et al. Standards for Reporting Qualitative Research: A Synthesis of Recommendations. *Academic Medicine* 2014;89(9):1245-51. doi: 10.1097/Acm.0000000000000388
32. Cohen-Mansfield J, Dakheel-Ali M, Marx MS, et al. Which unmet needs contribute to behavior problems in persons with advanced dementia? *Psychiatry research* 2015;228(1):59-64.
33. Brodaty H, Draper B, Low LF. Nursing home staff attitudes towards residents with dementia: strain and satisfaction with work. *J Adv Nurs* 2003;44(6):583-90. doi: 10.1046/j.0309-2402.2003.02848.x
34. Killett A, Hyde P, Burns D, et al. How organizational factors interact to influence the quality of care of older people in the care home sector. *Journal of Health Services Research & Policy* 2013;18(1_suppl):14-22. doi: 10.1177/1355819613476016
35. Stanyon MR, Griffiths A, Thomas SA, et al. The facilitators of communication with people with dementia in a care setting: an interview study with healthcare workers. *Age Ageing* 2016;45(1):164-70. doi: 10.1093/ageing/afv161
36. Lawrence V, Fossey J, Ballard C, et al. Helping staff to implement psychosocial interventions in care homes: augmenting existing practices and meeting needs for support. *Int J Geriatr Psychiatry* 2016;31(3):284-93. doi: 10.1002/gps.4322
37. Backhouse T, Killett A, Penhale B, et al. The use of non-pharmacological interventions for dementia behaviours in care homes: findings from four in-depth, ethnographic case studies. *Age Ageing* 2016;45(6):856-63. doi: 10.1093/ageing/afw136
38. Vernooij-Dasssen MJ, Faber MJ, Olde Rikkert MG, et al. Dementia care and labour market: the role of job satisfaction. *Aging Ment Health* 2009;13(3):383-90. doi: 10.1080/13607860902861043
39. Sormunen S, Topo P, Eloniemi-Sulkava U, et al. Inappropriate treatment of people with dementia in residential and day care. *Aging Ment Health* 2007;11(3):246-55. doi: 10.1080/13607860600963539

For peer review only - http://bmjopen.bmj.com/site/about/guidelines.xhtml

40. Cooper C, Marston L, Barber J, et al. Do care homes deliver person-centred care? A cross-sectional survey of staff-reported abusive and positive behaviours towards residents from the MARQUE (Managing Agitation and Raising Quality of Life) English national care home survey. *PLOS ONE* 2018;13(3):e0193399. doi: 10.1371/journal.pone.0193399

Legends for figures

Figure 1: Outline of recruitment and data collection procedures

For peer review only

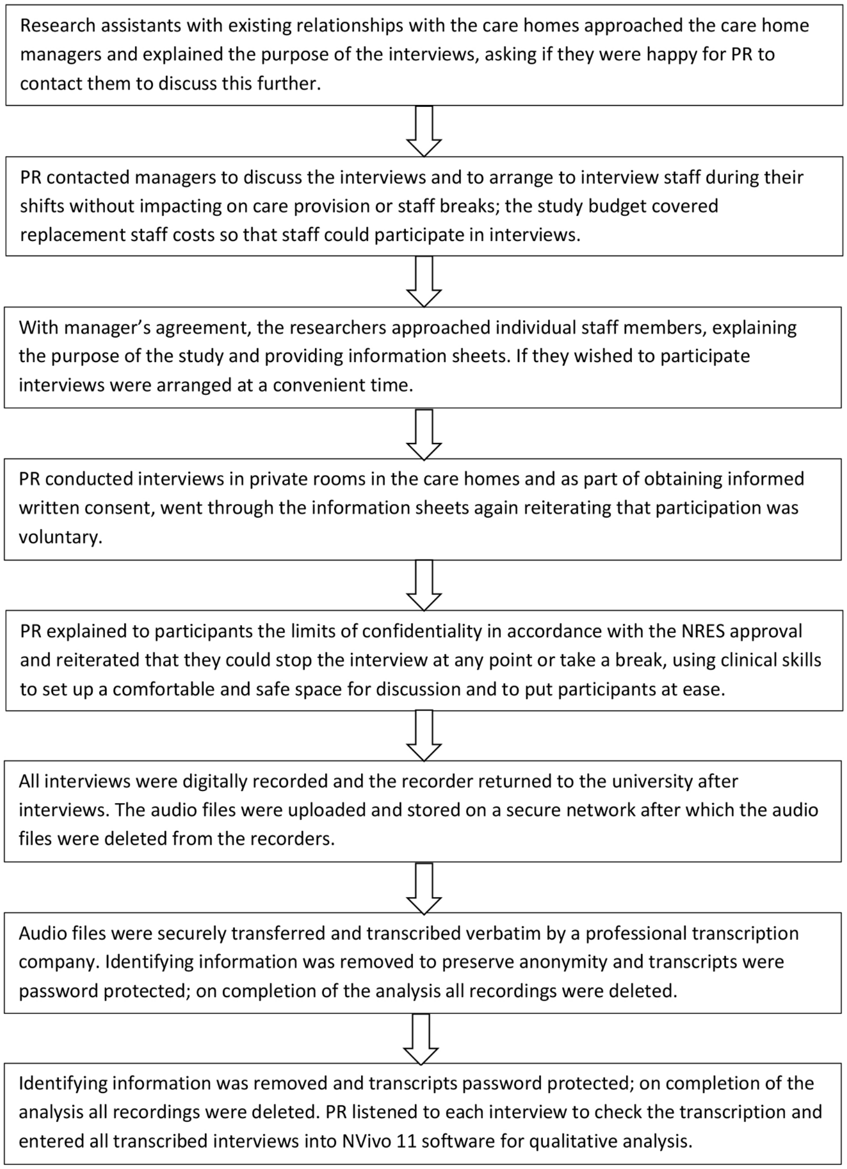

Figure 1: Outline of recruitment and data collection procedures

166x226mm (300 x 300 DPI)

UCL DIVISION OF PSYCHIATRY
FACULTY OF BRAIN SCIENCES
BLOOMSBURY CAMPUS

Study to improve agitation and quality of life for people with dementia in care homes: Care worker semi-structured interview schedule

Introductions: As you know *I am a researcher from University College London and I will be conducting and recording this interview. Everything you say is confidential but I would like you to introduce yourselves for the recording so that the typist can identify you. My name is….*

Description of the research: *Thank you for agreeing to take part in this interview, I am interested in how people working in care homes think about and cope with residents with agitation; when talking about agitation we are referring to behaviours such as restlessness, pacing, shouting and verbal or physical aggression. We are asking because we know that this is something you and your colleagues manage a lot and we want to make use of your expertise. I will be asking about your experiences of working with people with agitation; about what works and what does not and what helps or hinders.*

We will use the information to help us to develop an intervention to reduce agitation in people with dementia living in care homes and we are interested in how to make the intervention something which is practical and fits with day to day work in busy care homes.

Confidentiality: *This interview is going to be audio recorded; whatever you tell me will be anonymised for the purposes of the study so you or any other individuals you mention will not be identifiable. However if you do disclose any information that you or someone else is being harmed I will ask your permission to disclose the information to my supervisor. We respect confidentiality but cannot keep it a secret if anyone is being seriously harmed.*

If at any point during the interview you feel that you need to stop or leave the room please do tell me.

Experiences of agitation:
I want you to think of a resident you know well who has been agitated. You don't need to tell me their name.

Q. Can you tell me a bit about the person and describe their behaviour and what happened?
- *What do you think may have caused their agitation?*
- *What do you think it means when they behave in this way?*
- *What else have you noticed in other residents when they become agitated?*

(Prompt: If there is nothing much to say about this resident ask them to think of someone else)

Managing agitation:
Q. When you think about the person and situation you were just describing, how did you manage the situation?
- *What was tried?*
- *What worked well?*
- *What did not work well?*
- *What did you feel able to do at the time?*
- *What did you notice other people doing?*
- *Tell me about good ways of communication with people like this?*
- *How do decisions get made about what to do?*
- *What else have you seen work well in other situations?*

For peer review only - http://bmjopen.bmj.com/site/about/guidelines.xhtml

- *How do you think ________________ would make a difference (pain control, talking, sensory stimulation, music, activities, more communication)*
- *What would stop them working? What would make them work?*

Barriers and facilitators
Q. What makes it easier for you to manage agitation in residents?

- *What is it about you?*
- *What is it about your role?*
- *What is it about the residents / their families?*
- *What is it about your team?*
- *What is it about your managers?*
- *What is it about your organisation / employer?*
- *What is it about the media e.g. TV and newspapers?*

Q. What makes it harder for you to manage agitation in residents?

- *What is it about you?*
- *What is it about your role?*
- *What is it about the residents / their families?*
- *What is it about your team?*
- *What is it about your managers?*
- *What is it about your organisation / employer?*
- *What is it about the media e.g. TV and newspapers?*

Impact of agitation
Q. What impact does resident's agitated behaviour have upon staff?
- *What effect does it have on how you feel at work?*
- *What effect does it have upon what you feel able to do?*
- *How does it affect your team?*

Support
Q. When it is difficult, do staff get support?
- *Do you feel you can ask for help when you need it?*
- *What sort of support do you find helpful? Who from?*
- *How do you and your colleagues support each other?*
- *What gets in the way of asking for support?*

Training
Q. What training have you had to help you to manage when residents become agitated?

(If they can think of training):
- *What was most useful?*
- *What was least useful?*

If they cannot think of past training):

Q. What training do you think would help you to feel more able to manage agitation?
- *What do you think would help you to put what you learn into practice?*
- *What would make it harder to put what you learn into practice?*

(Prompt re self, role, team, managerial, organisational factors)

Other
Q. Before we finish, is there anything else you would like to mention that we have not already covered?

Would you be willing to give us feed back about any materials we may develop in future?

Thank you for taking part today.

For peer review only - http://bmjopen.bmj.com/site/about/guidelines.xhtml

What works in practice? How do care home staff understand and manage agitation in people with dementia

We are writing to you because you were interviewed by us some time ago about your experiences of caring for residents with dementia who experience agitation. We wanted to thank you again for taking part in our study and also to send you a summary of what we found out.

We interviewed 25 members of staff in six different care homes about what happens when residents become agitated, how you as staff manage and respond, and what makes it harder or easier to manage.

We have used what you and the other staff we interviewed told us about what works to develop a staff training intervention which we are currently testing in ten care homes, to see if it helps staff when residents become agitated. We have tried to address some of the things that staff told us makes it harder to manage agitation and have included some of the ideas about what can make it easier.

What agitated behaviours did staff experience?

Staff told us that they were coping with a range of agitated behaviours, the most common ones were verbal and physical aggression, like hitting or pinching (especially during personal care), screaming and getting upset, and repetitive behaviours like wandering up and down or repeating words.

Many staff members told us that these behaviours were often quite extreme, could get worse very quickly, were hard to predict and could last a long time. Staff also told us that agitation was different for different each person and 'no two residents are the same'.

How did agitation make staff feel?

Many staff said it could be difficult when residents become agitated, especially if it involves more than one resident. Staff sometimes felt frightened, especially when residents were aggressive. When nothing seemed to help, staff felt hopeless and like they did not have power to make things better. Some staff talked about how even though they tried to stay calm and not react badly, sometimes they felt angry or upset when residents were very agitated.

What did staff feel caused agitation?

Staff described how although it can sometimes feel like a 'guessing game' when trying to work out why someone is agitated, there were often a number of possible reasons for what may be triggering agitation. These included:

- Agitation as a sign of being upset
- Agitation as a sign of physical pain or discomfort
- Agitation as caused by how other people react
- Agitation as caused by the persons surroundings e.g. too hot, noisy, boring
- Agitation as caused by the dementia itself

For peer review only - http://bmjopen.bmj.com/site/about/guidelines.xhtml

What helps staff to prevent agitation from happening?

All the staff we spoke to said it was important to get to know the individuals in their care and build trust with the residents. There were three main things that staff said helped them to get to know residents and prevented the residents from becoming agitated. These were:

1. Seeing the person and not the disease

Well, I think they should be able to come in, yes, do the personal care, but while you're doing the personal care, look at the rest of the person, not only the bit you're washing and dressing, remember that they're a human being, remember that they've lived a life. (Unit manager)

2. Connecting with previously valued identities

Well, I always like to know what did you used to do in your time. What work do you like doing, you know. All the different things, really, in life… (Activities coordinator)

3. Playing along rather than correcting

While you standing arguing with someone saying, no, you're 90 and your kids are all grown up, to them they're not. They're still at school. They need help so we just say to them... so just go in their reality with them. If they're looking for their kids, the kids are at school. They're all right. (Deputy Manager)

4. Making people feel comfortable and at home

It's a 24 hour process and this is their home, they can get up when they like, as long as they eat and they feel comfortable, that's the most important. (Senior carer)

How do staff respond when residents are agitated?

Staff described the strategies they used when residents were agitated and how they tried to be flexible and use 'trial and error':

There's no hard and fast rules, it's just really sort of trying to read the situation really. (Care assistant)

For peer review only

Popular strategies included:

- Distracting and reassuring residents
- Giving agitated residents space
- Walking away

When these approaches did not work, staff would ask for help from senior colleagues and would sometimes get help from specialists outside of the care home. Some staff felt medication could help but said that often it had side effects and should only be used as a last resort.

What makes it harder or easier for staff to manage when residents are agitated?

1. Having the right approach makes it easier:

Staff described that being motivated, compassionate and caring, as well as recognising that you can only do your best, made it easier to do the job. They often compared this to feeling that some staff are 'only in it for the money'.

> *And what else gives us hope through the day? …I love my job, very much, and there is no explanation for that. You either have that or not. I could have done retail… I go through the day because I feel sorry for these people, simple as that. I… and I want to instinctively make it better. (Care assistant)*

2. Communication is key:

Staff saw clear communication between team members as important. To respond well to agitation, staff needed to feel that they were not on their own and that colleagues would help if needed. Staff also said that building a good relationship with the families of residents was very important.

> *You do sometimes … think to yourself, I don't know if I can cope with this today but then… you take five minutes, you have a chat with one of the other carers and then we sort it out. (Care assistant)*

3. Task focused vs person centred care:

Staff talked about not having enough time to spend with residents because of the staffing levels or because of all of the different jobs and tasks that they were expected to do during each shift. Staff said that they liked having 'hands on' managers who were approachable and that when they did not feel supported by management they would tend to just try and sort out problems themselves. Staff found it helpful to have good quality training but also said that learning on the job and from each other was very important.

For peer review only - http://bmjopen.bmj.com/site/about/guidelines.xhtml

I have had training [but] I've gone to management and they've taught me a different way to try and cope with it, I feel being there, dealing with it, doing it, is the best training. (Care assistant)

4. The care home industry makes it harder:

Staff talked about how television and newspapers always seem to focus on negative aspects of care, especially abuse and neglect, ignoring the good work that they saw happening and the impact of aggression from residents towards staff. This meant that families were more worried about their relatives living in a care home and staff felt this made them more worried about making mistakes or getting into trouble at work.

Sometimes it would be so lovely to hear a nice story about dementia, and staff, and what people do, and ... you don't hear things about residents lashing out at carers. (Care assistant)

Staff in all types of home talked about some of the financial problems in care homes and how they felt that focusing on business and profit made it harder for them to meet the needs of individuals.

Sometimes it can be challenging because if the budget doesn't meet ... then the staff need to be reduced, and ... the needs of the residents take second place. (Deputy Manager)

We would like to thank you again for taking part. We are also in the process of writing an article based on these findings so that a wide range of health and social care professionals will be able to learn from it.

If you have any thoughts or comments on this summary we would really appreciate your feedback. We are interested in hearing if you feel there is anything missing from this summary and whether it fits well or does not fit with your own experiences. You can either email xxx or write using the enclosed stamped addressed envelope.

For peer review only - http://bmjopen.bmj.com/site/about/guidelines.xhtml

Standards for Reporting Qualitative Research (SRQR)*

http://www.equator-network.org/reporting-guidelines/srqr/

Title and abstract

	Page/line no(s).
**Title** - Concise description of the nature and topic of the study Identifying the study as qualitative or indicating the approach (e.g., ethnography, grounded theory) or data collection methods (e.g., interview, focus group) is recommended	Page 1 line 1
**Abstract** - Summary of key elements of the study using the abstract format of the intended publication; typically includes background, purpose, methods, results, and conclusions	Page 2 line 1

Introduction

**Problem formulation** - Description and significance of the problem/phenomenon studied; review of relevant theory and empirical work; problem statement	Page 3 lines 1-41
**Purpose or research question** - Purpose of the study and specific objectives or questions	Page 3 lines 37-41

Methods

**Qualitative approach and research paradigm** - Qualitative approach (e.g., ethnography, grounded theory, case study, phenomenology, narrative research) and guiding theory if appropriate; identifying the research paradigm (e.g., postpositivist, constructivist/ interpretivist) is also recommended; rationale**	Page 4 line 25
**Researcher characteristics and reflexivity** - Researchers' characteristics that may influence the research, including personal attributes, qualifications/experience, relationship with participants, assumptions, and/or presuppositions; potential or actual interaction between researchers' characteristics and the research questions, approach, methods, results, and/or transferability	Page 1 lines 9-10<br>Page 4 lines 25-30
**Context** - Setting/site and salient contextual factors; rationale**	Page 4 lines 2-16
**Sampling strategy** - How and why research participants, documents, or events were selected; criteria for deciding when no further sampling was necessary (e.g., sampling saturation); rationale**	Page 4 lines 2-16<br>Figure 1
**Ethical issues pertaining to human subjects** - Documentation of approval by an appropriate ethics review board and participant consent, or explanation for lack thereof; other confidentiality and data security issues	Page 4 lines 3-4<br>Figure 1
**Data collection methods** - Types of data collected; details of data collection procedures including (as appropriate) start and stop dates of data collection and analysis, iterative process, triangulation of sources/methods, and modification of procedures in response to evolving study findings; rationale**	Page 4 lines 9-<br>page 5 line 2<br>Figure 1

For peer review only - http://bmjopen.bmj.com/site/about/guidelines.xhtml

**Data collection instruments and technologies** - Description of instruments (e.g., interview guides, questionnaires) and devices (e.g., audio recorders) used for data collection; if/how the instrument(s) changed over the course of the study	Page 4 lines 9-page 5 line 2<br>Figure 1<br>Supplementary appendix 1
**Units of study** - Number and relevant characteristics of participants, documents, or events included in the study; level of participation (could be reported in results)	Page 5 lines 4-8<br>Table 1
**Data processing** - Methods for processing data prior to and during analysis, including transcription, data entry, data management and security, verification of data integrity, data coding, and anonymization/de-identification of excerpts	Figure 1<br>Page 4 lines 25 – page 5 line 2
**Data analysis** - Process by which inferences, themes, etc., were identified and developed, including the researchers involved in data analysis; usually references a specific paradigm or approach; rationale**	Page 4 lines 25 – page 5 line 2
**Techniques to enhance trustworthiness** - Techniques to enhance trustworthiness and credibility of data analysis (e.g., member checking, audit trail, triangulation); rationale**	Page 4 lines 25 – page 5 line 2<br>Supplementary appendix 2

Results/findings

**Synthesis and interpretation** - Main findings (e.g., interpretations, inferences, and themes); might include development of a theory or model, or integration with prior research or theory	Page 5 line 23-page 11 line 22<br>Supplementary appendix 4
**Links to empirical data** - Evidence (e.g., quotes, field notes, text excerpts, photographs) to substantiate analytic findings	Page 5 line 23-page 11 line 22

Discussion

**Integration with prior work, implications, transferability, and contribution(s) to the field** - Short summary of main findings; explanation of how findings and conclusions connect to, support, elaborate on, or challenge conclusions of earlier scholarship; discussion of scope of application/generalizability; identification of unique contribution(s) to scholarship in a discipline or field	Page 11 line 24 – page 12 line 25
**Limitations** - Trustworthiness and limitations of findings	Page 12 line 32-40

Other

**Conflicts of interest** - Potential sources of influence or perceived influence on study conduct and conclusions; how these were managed	Page 14 line 17-18
**Funding** - Sources of funding and other support; role of funders in data collection, interpretation, and reporting	Page 14 line 7-16

*The authors created the SRQR by searching the literature to identify guidelines, reporting standards, and critical appraisal criteria for qualitative research; reviewing the reference lists of retrieved sources; and contacting experts to gain feedback. The SRQR aims to improve the transparency of all aspects of qualitative research by providing clear standards for reporting qualitative research.

For peer review only - http://bmjopen.bmj.com/site/about/guidelines.xhtml

**The rationale should briefly discuss the justification for choosing that theory, approach, method, or technique rather than other options available, the assumptions and limitations implicit in those choices, and how those choices influence study conclusions and transferability. As appropriate, the rationale for several items might be discussed together.

Reference:

O'Brien BC, Harris IB, Beckman TJ, Reed DA, Cook DA. **Standards for reporting qualitative research: a synthesis of recommendations.** *Academic Medicine*, Vol. 89, No. 9 / Sept 2014
DOI: 10.1097/ACM.0000000000000388

For peer review only

For peer review only - http://bmjopen.bmj.com/site/about/guidelines.xhtml

Appendix 4: Table of themes

Overarching themes	Sub themes
1. Behaviours expressing unmet need	1.1 Unmet physical need<br>1.2 Unmet emotional need<br>1.3 Unmet environmental need
2. Staff emotional responses to agitation	2.1 Feeling powerless and disheartened<br>2.2 Feeling frightened<br>2.3 Trying not to react
3. Understanding the individual helps	3.1 Seeing the person not the disease<br>3.2 Connecting with previously valued identities<br>3.3 Playing along with rather than correcting<br>3.4 Making people feel comfortable and at home
4. Constraints on staff responses	4.1 Procedural constraints<br>4.2 Structural constraints<br>4.3 Support and training<br>4.4 Culture of fear and scrutiny

For peer review only
